# Breakthrough in architecture—Contemporary Iraqi architecture case study

**DOI:** 10.1371/journal.pone.0355155

**Published:** 2026-08-31

**Authors:** Fawzia Irhayyim Al-Assadi, Osamah Abdulmunem Al-Tameemi, Aya F. Alobaidi

**Affiliations:** Department of architecture, University of Baghdad, Baghdad, Iraq; Imam Ja’afar Al-Sadiq University, IRAQ

## Abstract

This research explores the concept of “architectural breakthrough “ as an intellectual and design phenomenon capable of transcending traditional patterns and redefining the relationship between form, function, and meaning in contemporary Iraqi architecture. This research aims to construct a clear theoretical framework that explains the mechanisms of architectural breakthrough and its role in generating innovation and strengthening spatial identity within post-2003 Iraqi contexts. The research employs a qualitative, analytical, and comparative approach, integrating field observation, document analysis, and semi-structured interviews, which were systematically analyzed using reflexive thematic analysis supported by NVivo 14 software, across three key case studies representing models of Iraqi architecture after 2003: the General Secretariat of the Council of Ministers building, the Chinese schools in Iraq, and the Central Bank of Iraq building. The findings demonstrate that architectural breakthrough is not limited to form but also encompasses cultural and social dimensions, serving as a means of reproducing identity by integrating technology and modernity with local values. Based on the empirical results, the research proposes a theoretical framework that links breakthrough, innovation, and transformation as mechanisms for revitalizing Iraqi architecture within a global context.

## Introduction

Contemporary Iraqi architecture serves as a profound indicator of the nation’s complex historical trajectory, embodying the dynamic interplay between cultural heritage, socio-political transformation, and the forces of globalization. In the post-2003 era, this architecture has been engaged in a critical renegotiation of national identity, attempting to reconcile a rich historical legacy with the demands of modernity, reconstruction, and global integration. Within this transformative context, the concept of an “architectural breakthrough”–a decisive design act that transcends prevailing paradigms to establish new frameworks for spatial thought and production–emerges as a potent mechanism for cultural and urban renewal. The scholarly discourse on modern Iraqi architecture has grown considerably, with studies examining its historical evolution, stylistic influences, and socio-political dimensions [1,2]. However, a significant theoretical lacuna persists. While existing literature often documents stylistic changes or catalogues new buildings, it largely lacks a systematic conceptual framework that specifically interrogates the mechanism of breakthrough. Prevailing studies tend to describe what has changed in the built environment but fall short of critically analyzing how transformative change is conceived, executed, and validated as a “breakthrough” within the Iraqi context. This gap is particularly evident in the post-2003 period, a time marked by an urgent search for a new architectural narrative. As noted by Al-Saadi [3], discussions on innovation in Iraqi architecture often remain anecdotal or focused on individual projects, without linking them to a broader theory of transformative change. Similarly, analyses of identity, such as those by Hamdy and Alasadi

[1] and Bassim & Alobaydi [4], adeptly trace symbolic references but seldom position these designs within a paradigm of innovation that challenges the status quo. Consequently, the intellectual and practical processes through which Iraqi architecture can rupture entrenched patterns, redefine the relationship between form and cultural meaning, and generate a sustainable, locally grounded modernity remain under-explored. This omission is critical without a clear framework to understand breakthroughs, the discourse risks interpreting bold new forms as isolated anomalies or superficial stylistic shifts, rather than as intentional, theorized acts of cultural and spatial redefinition with the potential to steer the development trajectory of the national built environment. Therefore, this research aims to address this identified gap directly. Its primary objective is to construct and apply a clear theoretical and analytical framework that explains the mechanisms of architectural breakthrough and its role in generating innovation and strengthening spatial identity in contemporary Iraq. To achieve this, the study pursues the following specific research questions: How can the concept of “architectural breakthrough” be theoretically defined and operationalized into measurable indicators within the context of contemporary Iraqi architecture? In what ways do select landmark projects in post-2003 Baghdad manifest breakthroughs across the dimensions of design innovation, sustainability, cultural identity, and functional quality? By examining these questions through an in-depth, qualitative analysis of three key case studies–the General Secretariat of the Council of Ministers, the Chinese Schools, and the Central Bank of Iraq–this research seeks to move beyond descriptive cataloguing. It aims to provide a critical lens for understanding how breakthroughs occur, what forms it takes, and what their implications are for the future of architectural production and cultural expression in Iraq and similar post-conflict, identity-seeking contexts.

## Theoretical framework

This section focuses on the key theoretical concepts directly relevant to the analytical framework and omits peripheral discussions to maintain conceptual clarity and alignment with the study objectives.

### Architectural breakthrough: Conceptual foundations and relation to innovation

While innovation is a widely discussed concept in architectural discourse, this study adopts “architectural breakthrough” as its primary analytical focus. Breakthrough is understood as a higher-order condition that transcends incremental innovation, representing a qualitative shift in architectural thinking, production, and impact. Accordingly, innovation is treated in this study as one of the enabling dimensions of breakthrough rather than its equivalent.

Architectural breakthrough and innovation are closely related yet conceptually distinct. While innovation often refers to the introduction of new ideas, techniques, or forms, breakthrough denotes a deeper transformative shift that redefines existing paradigms and establishes new directions in architectural thought and practice. In this sense, breakthrough operates as a cumulative and integrative condition in which multiple forms of innovation converge to produce a significant qualitative change.

Existing literature addressing architectural breakthroughs remains limited and often implicit, with most studies focusing instead on innovation as a driver of architectural transformation. Therefore, this review draws selectively on key studies of innovation to support the conceptualization of breakthrough, while maintaining a clear distinction between the two concepts. These studies highlight how the recombination of technical, environmental, and aesthetic strategies can lead to paradigm shifts in architecture, contributing to new relationships between form, function, and cultural expression [2]. This review explores the most prominent theses that have addressed innovation as a driving force for disrupting prevailing paradigms and producing new concepts in architectural thought:

**Feizbahr and Pourzanjani, in The Evolution of Architectural Styles: From Modernism to Postmodernism [5]**, highlight modern architecture as a major shift in global design history. Moving away from traditional methods, architects embraced innovation and scientific principles, creating a globally recognized style focused on logic and material efficiency, in contrast to pre-modernist styles of the Industrial Revolution.

**In their 1990 study Architectural Innovation: The Reconfiguration of Existing Product Technologies and the Failure of Established Firms, Rebecca M. Henderson and Kim B. Clark [6]** A cognitive model of innovation defines products as systems of interconnected components and identifies four types: incremental, modular, architectural, and radical. Radical innovation involves entirely new designs with novel components and configurations, often leading to breakthroughs that disrupt markets and establish new industry standards.

**The book “In Modern Architecture -the Museum of Modern Art: by Arthur Drexler, (1979)** [7] Modern architecture, from the 20th century to the 1980s, moved beyond traditional styles by embracing innovation, technology, and new materials to shape a modern environment. Drexler highlights key works and theories that influenced this shift, emphasizing their impact on cities and society. The movement strongly reflects the relationship between form and function.

**Theoretical criticism of architectural Breakthrough: by (Nicholas Bullock, 2009) [8].** Theoretical critiques of breakthrough in contemporary architecture note that some designs may neglect cultural and social context, appearing disconnected from their surroundings. However, these studies emphasize breakthrough ’s potential to balance heritage and modernity, promote sustainability, and enhance social interaction, offering valuable insight into the challenges and opportunities for architects in modern Iraqi architecture.

### Breakthrough aspects in general

1. **Cultural Breakthrough:** refers to the impact of a cultural idea or element on society resulting in changes in values, customs, and traditions.
2. **Intellectual Breakthrough:** Introducing new critical or philosophical concepts that undermine established knowledge in design. [9].
3. **Cognitive Breakthrough:** Expanding the horizons of perception and reinterpreting the relationships between matter, space, and meaning.
4. **Social Breakthrough:** Changing the nature of the interaction between humans and public space or the built environment [10].

*The research, therefore, arrives at a procedural definition of breakthrough as presenting something remarkably new that radically transforms a particular field, such as thought, philosophy, or production, by innovatively and effectively breaking existing traditional rules. The breakthrough often occurs suddenly.*

Research considers that innovation and breakthrough can be simultaneous processes, as innovation need to breakthrough to achieve a radical impact in some cases. The research will explore the concept of breakthroughs in architecture.

### Breakthrough in architecture

In architecture, “breakthrough” extends beyond physical structures, influencing cultural and social dynamics by redefining norms and interactions. Closely linked to experimental architecture, it encourages bold, unconventional designs that challenge traditional methods and inspire societal change [11]. Studies emphasize the brain’s engagement with design, highlighting creativity as a tool for innovation. While [9] defines creativity as a contextual and cognitive advancement, [12] focuses on originality and practical relevance. [13] views breakthrough through contrasts and deconstruction, and [14] sees it as a driver of urban identity and improved quality of life through innovative architectural strategies.

### Breakthrough and change in architecture

Constancy describes a condition in which something stays the same over time, while change refers to shifting from one state to another by altering, removing, or adding elements. This transformation creates new connections between existing components and the forces behind the change, whether those elements are physical or conceptual [15]

#### The speed of change.

In it, the changes that occur to architectural identity can be studied in terms of the period that the change takes to occur:

1 **Gradual change:** Traditional architecture developed slowly over long periods, shaped by consistent social, economic, cultural, and political influences. Some architectural models proved effective in responding to environmental needs and were repeated over time, eventually becoming standard designs. This gradual evolution reflects how buildings naturally took shape through the influence of tradition and the passage of time [16].

*The* research *considers that this type of change does not constitute a breakthrough in architecture; it is regarded as a natural consequence of environmental factors, occurring sequentially. It is important to understand how these factors interact with architecture’s structure and their gradual impact on it*.

**Fig 1:**
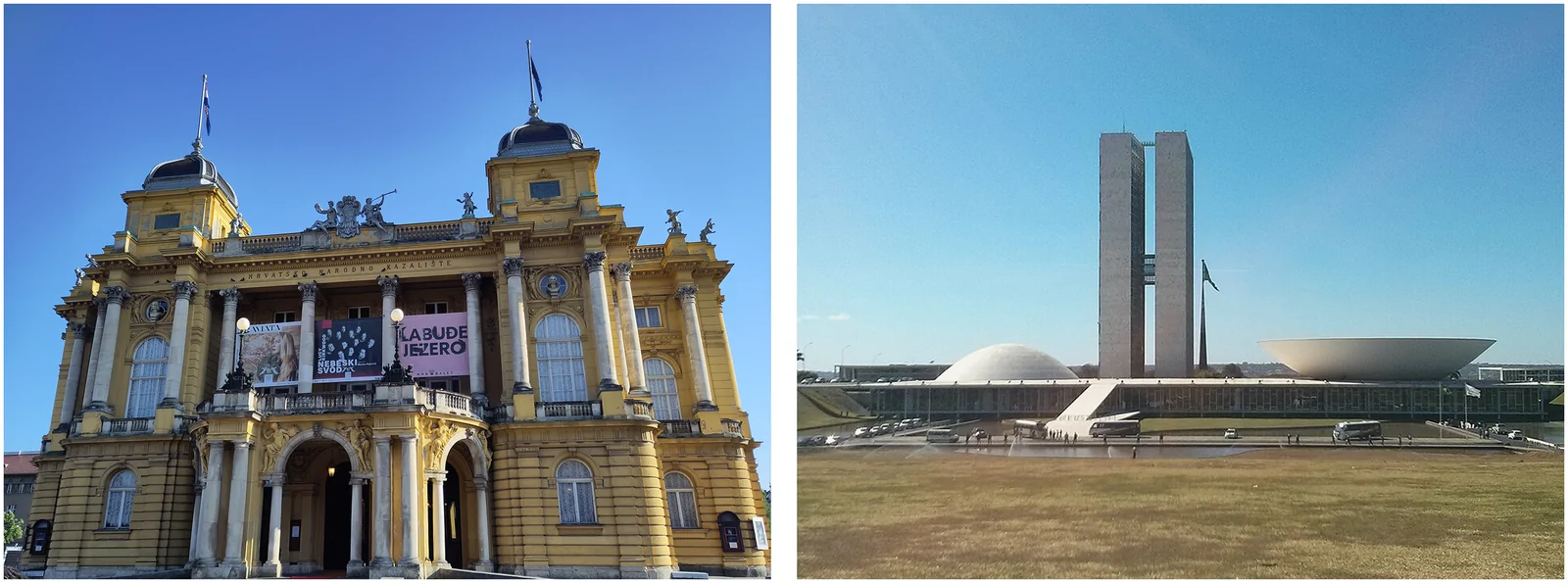
Selected examples of iconic architectural buildings. **(A)** National Opera of Zagreb, Zagreb, Croatia, designed by Fellner & Helmer. Photograph by Alex Sirac, via Wikimedia Commons, licensed under CC BY 4. **(B)** National Congress of Brazil, Brasília, Brazil, designed by Oscar Niemeyer. Image by Alex98, available under CC0 1.0 Public Domain Dedication via Wikimedia Commons.

2 **Sudden change:** Modern architecture represented a sharp departure from traditional design, driven by major intellectual, scientific, and economic shifts. Emphasizing simplicity, functionality, and industrial materials like glass, steel, and concrete, it rejected ornamentation and classical styles. Architects such as Le Corbusier embodied these principles through ideas like the “Five Points of Architecture,” promoting efficiency, open plans, and minimalist design [17] (Fig 1) some of iconic architectural buildings.

The research believes that this type of change is what causes breakthroughs in architecture because of the speed of change, in addition, it often arises after a revolution in thought, scientific progress, or economic change, and these revolutions are what cause the breakthrough.

**Fig 2:**
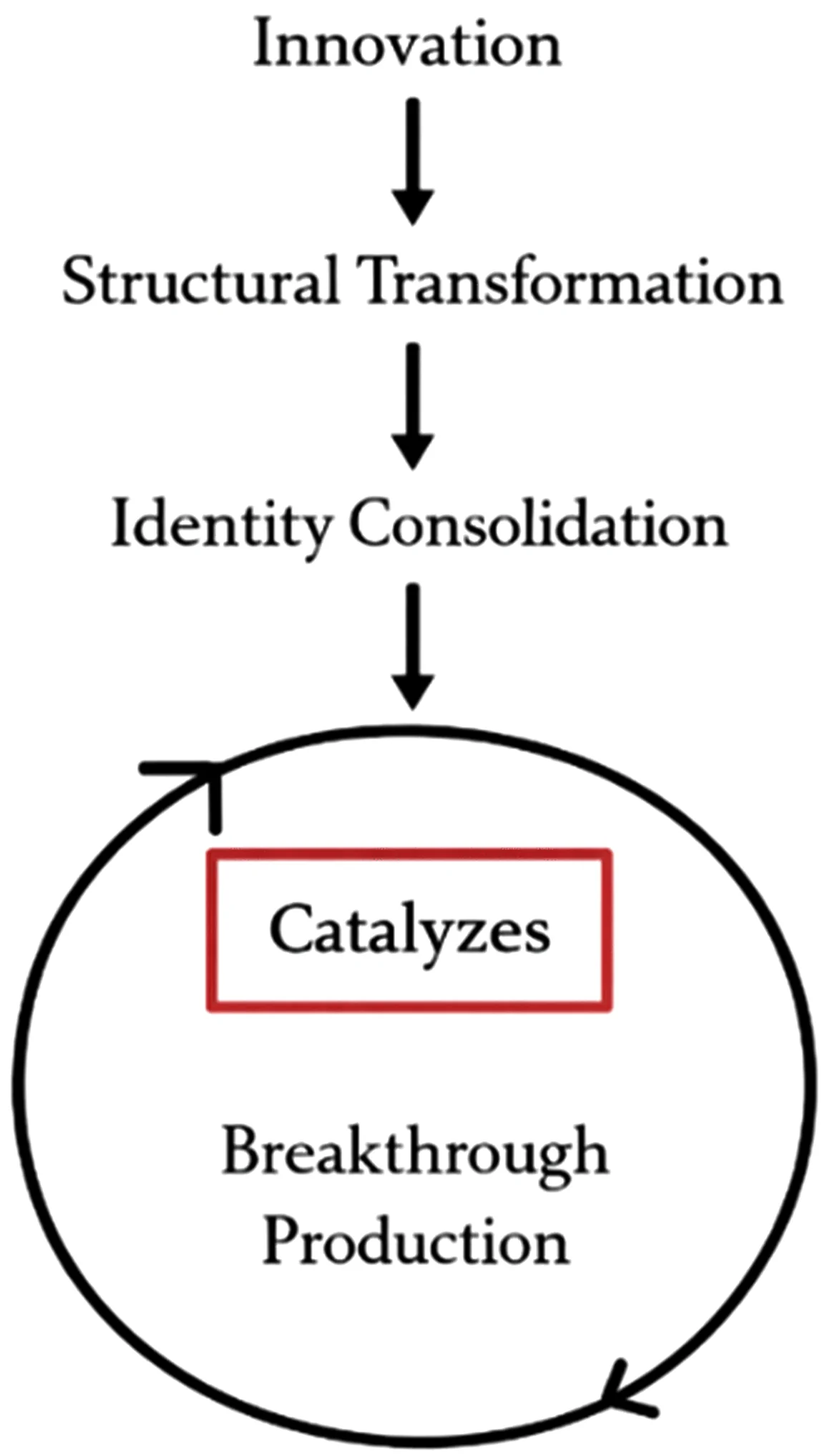
Theoretical feedback model of breakthrough production in architecture. Created by the authors.

Breakthroughs in architecture represent creativity and innovation, emphasizing originality and quality design. It drives new solutions to real-world challenges by breaking repetitive patterns and blending artistic imagination with thoughtful design. **Based on the above, the research concludes that breakthroughs are linked to imagination, creativity, innovation, and thus change.** Breakthrough and its relationship to innovation, imagination, and change will be addressed within the context of the research. (Fig 2) Illustrates the feeder cycle in which innovation begins, is transformed into applications and practices (transformation), and is integrated into the local identity, thus feeding the opportunities for breakthrough and dissemination.

### Applied levels of breakthrough on the architectural side

**Fig 3:**
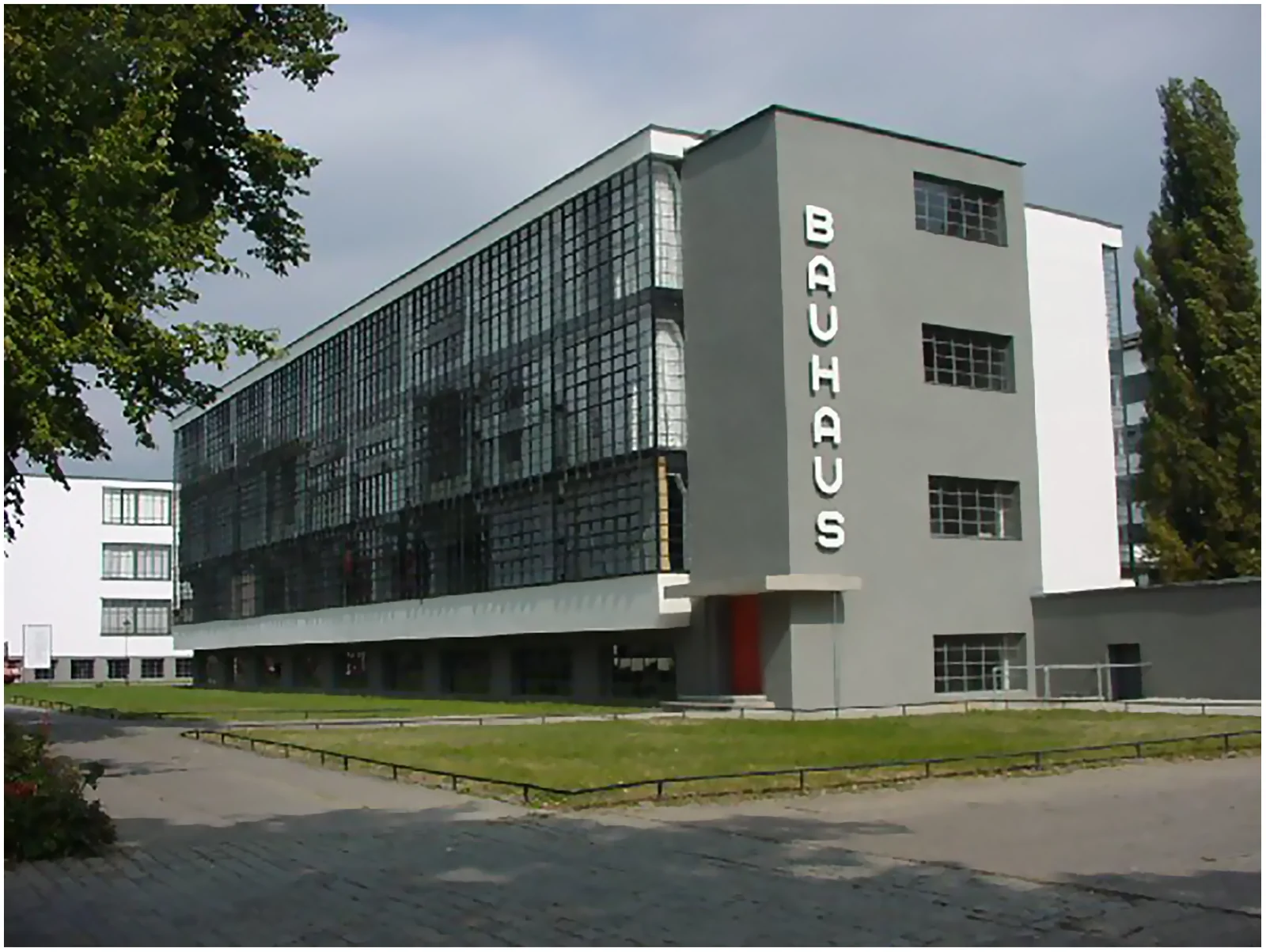
Bauhaus Dessau main building (1925–1926), Dessau, Germany. Designed by Walter Gropius. *Source: Alper.87, “Bauhaus” (Wikimedia Commons). This work has been released into the public domain by the copyright holder and may be used without restriction.* https://commons.wikimedia.org/w/index.php?title=File:Bauhaus.1993.jpg&oldid=818789007.

Architectural breakthroughs occur on various levels—intellectual, production, experiential, and physical—enabling architects to reshape building design with meaningful impact. Intellectually, this involves embracing new ideas, as seen in Modernism, which values simplicity and function. Walter Gropius Bauhaus Dessau Building in Germany is a key example of this approach, as shown in (Fig 3) Show Bauhaus Dessau main building in Germany [18]

**Fig 4:**
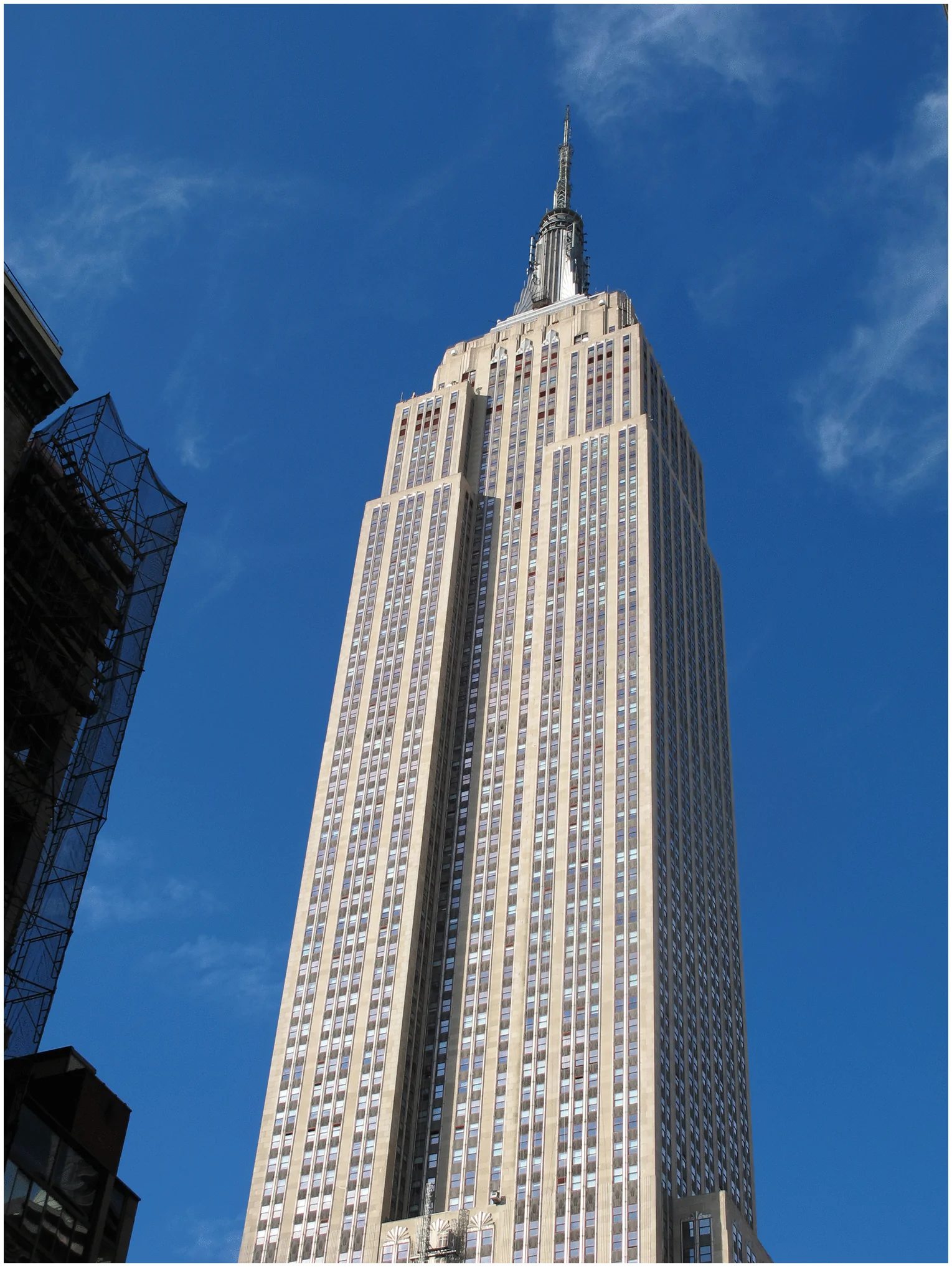
Empire State Building, New York City, USA. Designed by Shreve, Lamb & Harmon. Photograph by Acediscovery, via Wikimedia Commons, licensed under CC BY 4.0.

At the product level, reference is made to actual architectural works that represent new development trends through innovative uses of materials and techniques. A prominent example is the **Empire State Building (1930)**, which demonstrates a major evolution in the use of steel and glass in skyscraper construction, as illustrated in (Fig 4) [19]

**Fig 5:**
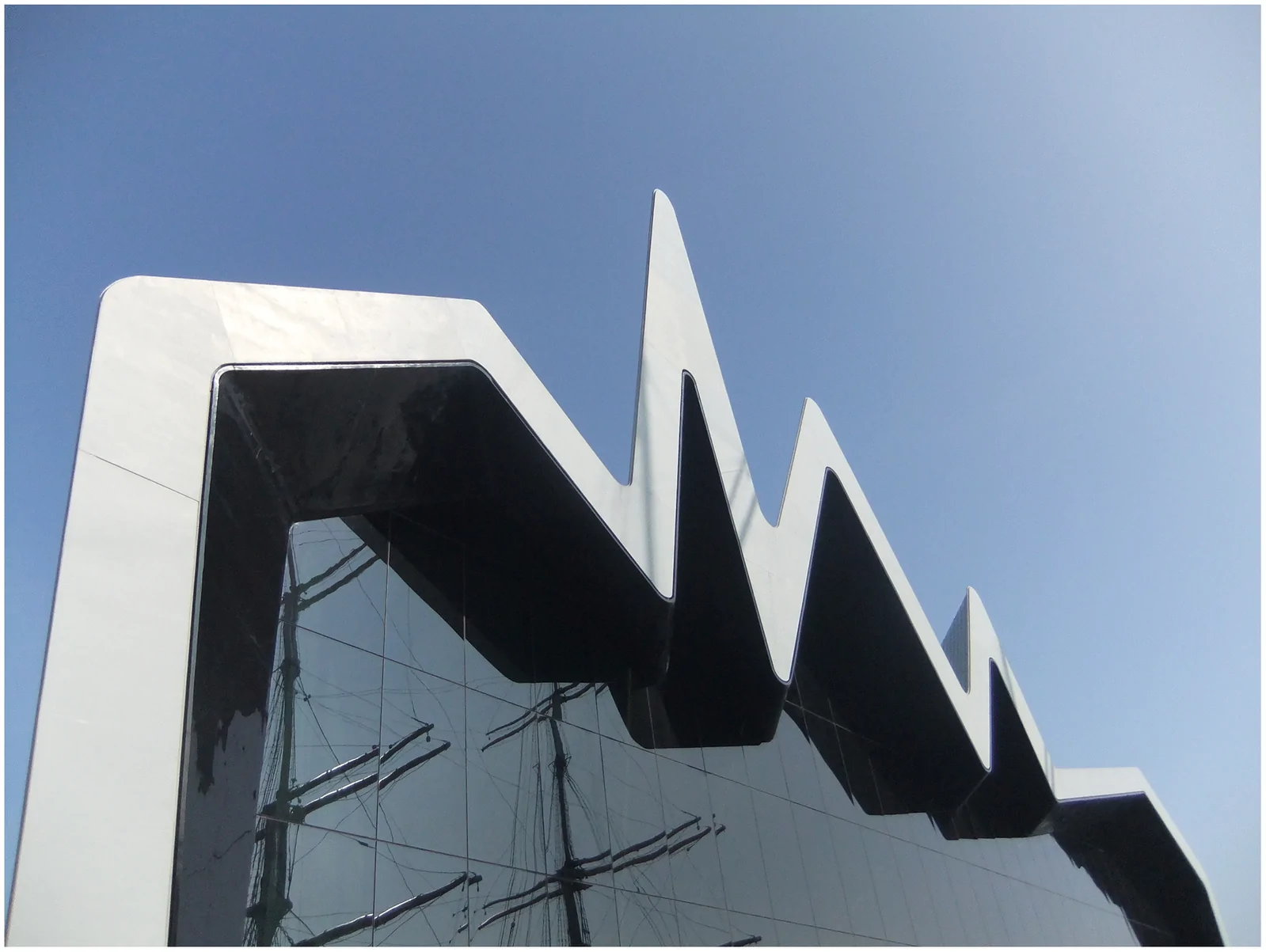
Riverside Museum, Glasgow, United Kingdom. Designed by Zaha Hadid. Photograph by Alex Livet (2012), originally published on Flickr and dedicated to the public domain under CC0 1.0.

At the embodiment level, this involves applying architectural concepts using new materials and techniques to realize specific design goals. Parametric architecture, as seen in Zaha Hadid’s Glasgow Riverside Museum, uses advanced software to create complex, non-traditional forms, (Fig 5) shows the building of the Riverside Museum, Glasgow, United Kingdom. [20]

**Fig 6:**
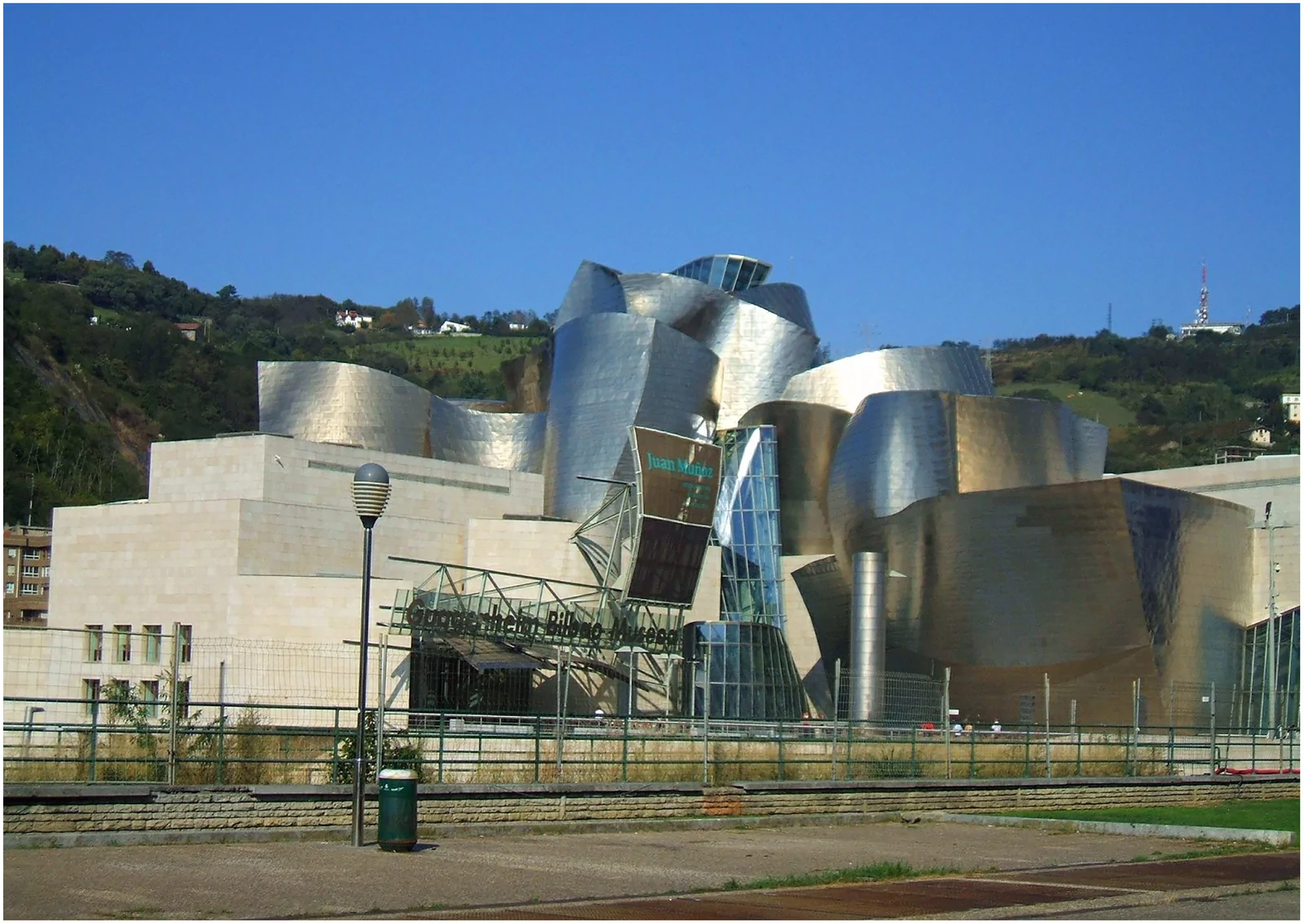
Guggenheim Museum Bilbao (1997), Bilbao, Spain. designed by Frank Gehry. *Source: Zarateman, “Museo Guggenheim Bilbao” (2010), Wikimedia Commons. Licensed under Creative Commons CC0 1.0 Universal Public Domain Dedication (Public Domain).* https://commons.wikimedia.org/w/index.php?title=File:Bilbao_-_Guggenheim_41.jpg&oldid=1187246937.

At the individual experience level, it refers to architects’ personal journeys that move beyond the familiar, offering fresh perspectives on design and construction. Frank Gehry’s work, like the Guggenheim Museum Bilbao (1997), Bilbao, Spain, exemplifies this through its innovative, flowing, and abstract forms, (Fig 6) Breaking with convention in the design of the Guggenheim Museum building in Bilbao. [21]

Creativity and breakthroughs are closely linked in architectural thought, especially when considering innovation’s role in driving change. [3,22]

**Fig 7:**
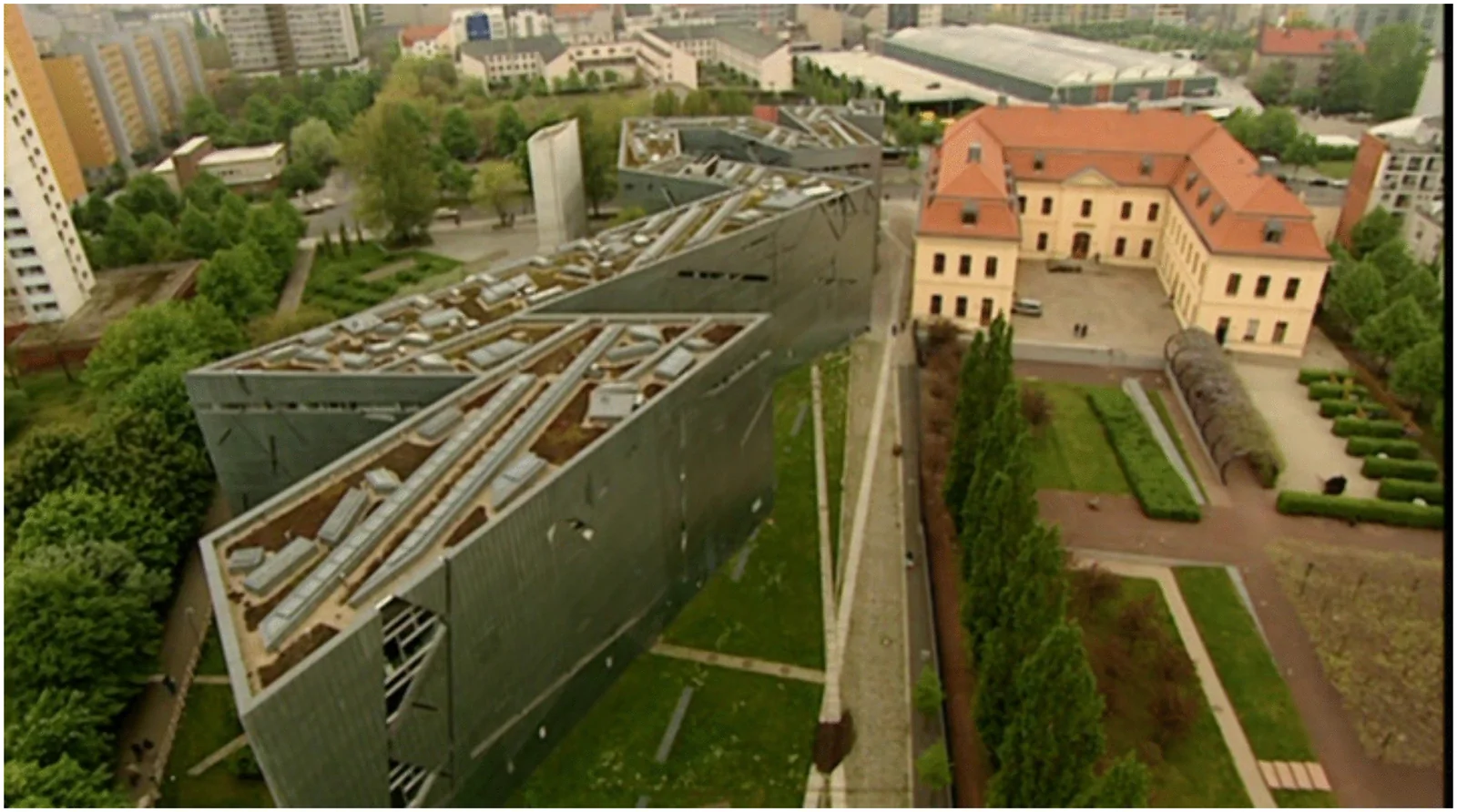
Jewish Museum, Berlin, Germany. Designd by Daniel Libeskind. Photograph by DDZ (2007), Image released into the public domain by the copyright holder.

The concept of “breakthrough “ is prominently featured in deconstructivism architecture, which challenges classical geometric principles. Architect Daniel Libeskind exemplifies this by dismantling traditional forms and reconfiguring them in unconventional, artistic ways, (Fig 7) Challenging classical architecture principles through deconstruction [23]

Larson discusses “breakthrough production” in architecture as a driver of sustained innovation. She highlights three essential factors:

1. Timing, that is, when the timing of new and unique ideas and solutions is appropriate to bring about change within the context.
2. Presence and ability to discriminate by elite practitioners to recognize and support their unique advantages before sponsoring institutions and recipients.
3. Potential of breakthrough for derivatives and subsequent models that may belong to the designer himself or others [24].

**Fig 8:**
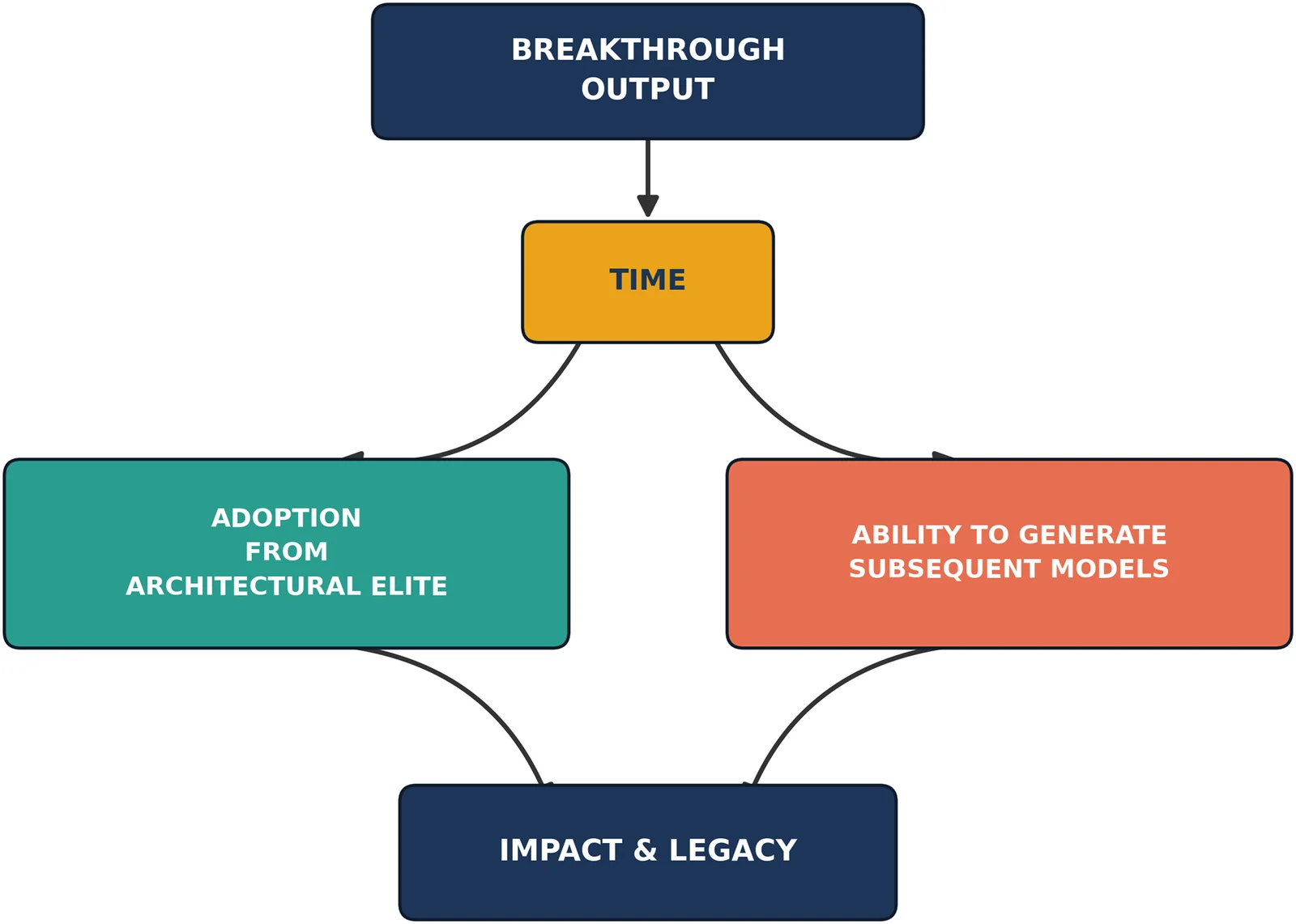
Factors contributing to the success of the breakthrough production in architecture. – create by Authors.

Breakthroughs in architecture refer to challenging traditional design norms and exploring innovative, unconventional solutions. It involves using non-traditional geometry and techniques to express bold, original ideas, which can significantly affect the built environment, both positively and negatively. The factors contributing to the success of breakthrough production in architecture can be illustrated in (Fig 8).

### Design orientations to achieve breakthroughs in architecture

**Fig 9:**
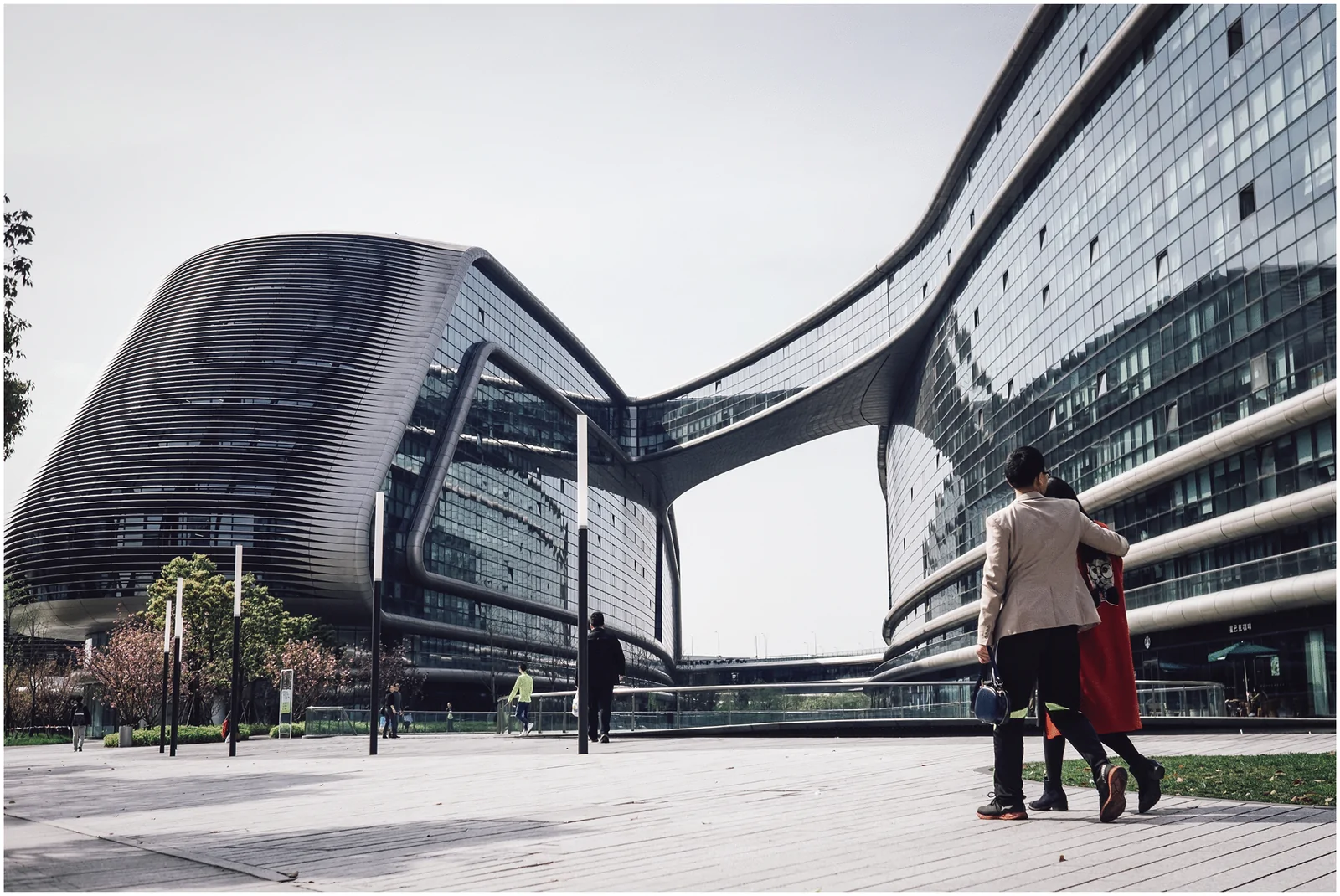
Sky SOHO, Hongqiao, Shanghai, China. Designed by Zaha Hadid. Photograph by Adi Constantin (2016), originally published on Unsplash under CC0 1.0 Public Domain Dedication.

**Fig 10:**
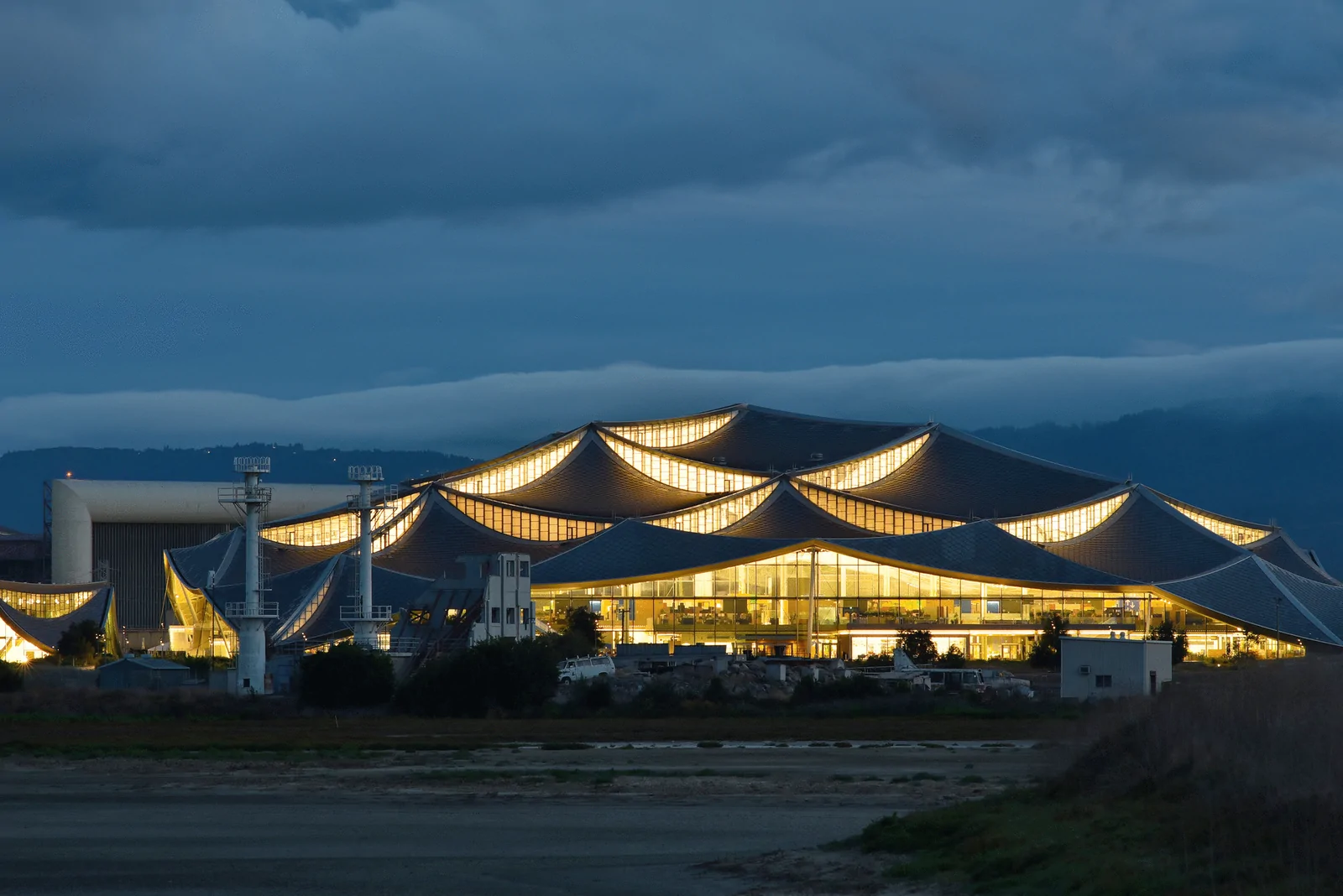
Google Bay View Campus, Mountain View, California. Designed by BIG (Bjarke Ingels Group) and Heatherwick Studio. Photo by Ian Abbott, via Flickr, licensed under CC BY 4.0.

- **Bold and unconventional design:** The breakthrough in architecture includes the use of unconventional design methods that defy expectations and exceed existing models. As in Zaha Hadid’s designs. (Fig 9) is show the use of unconventional design techniques is evident.

- **Using innovative materials:** Breakthrough can be shown through the use of new materials or innovative building techniques, resulting in unique structures such as materials that can produce energy such as colored solar cells which are used as interface packaging materials as well as clean electricity production, as Google Bay View Campus building, the building was equipped with 50,000 silver solar panels integrated into the roof envelope, as illustrated in (Fig 10). [25,26]

**Fig 11:**
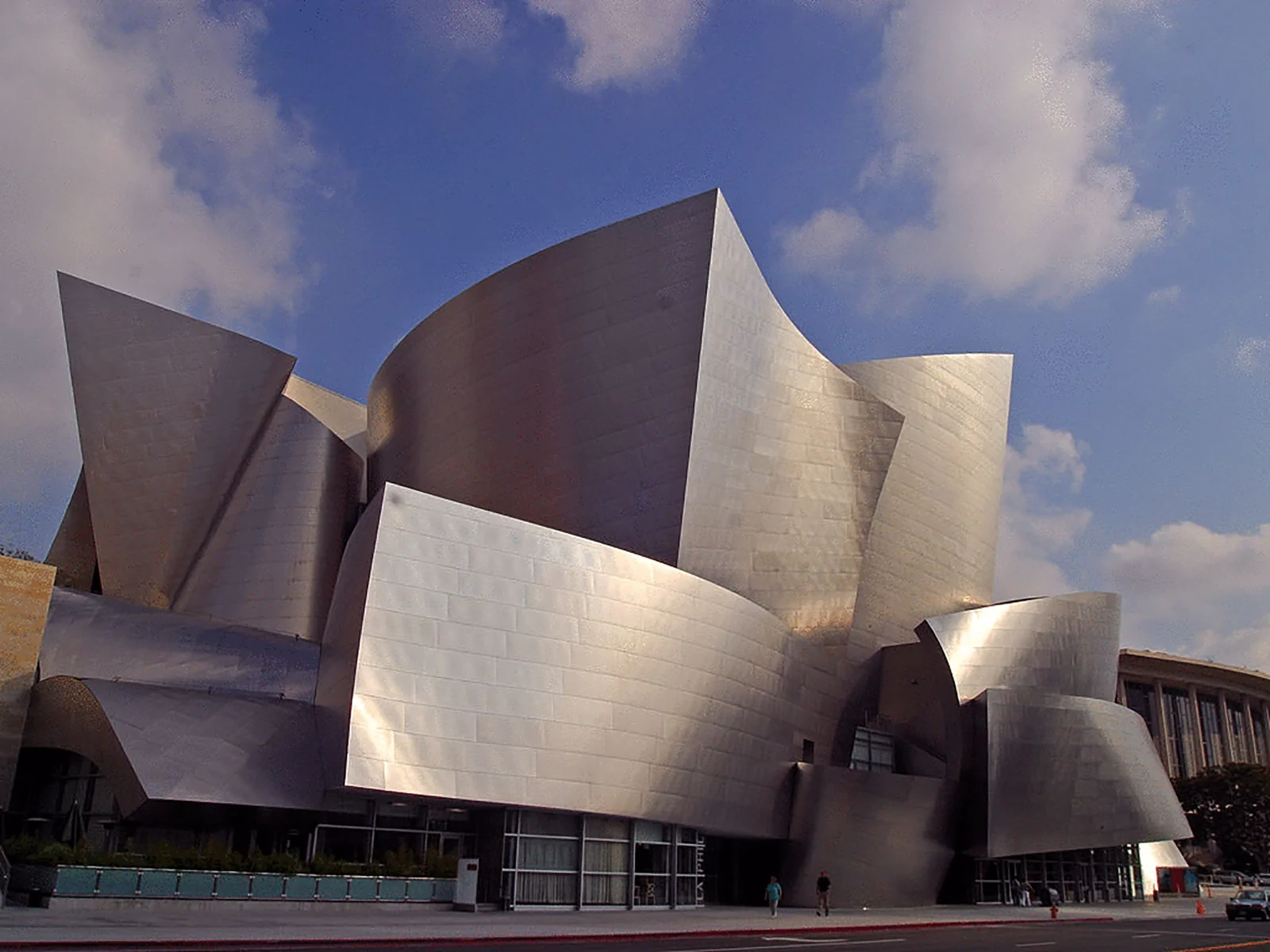
Iconic examples of deconstructivist and high-tech architecture. Walt Disney Concert Hall, Los Angeles, USA, designed by Frank Gehry (2003). Photograph by Jon Sullivan, via PDPhoto.org, public domain.

**Fig 12:**
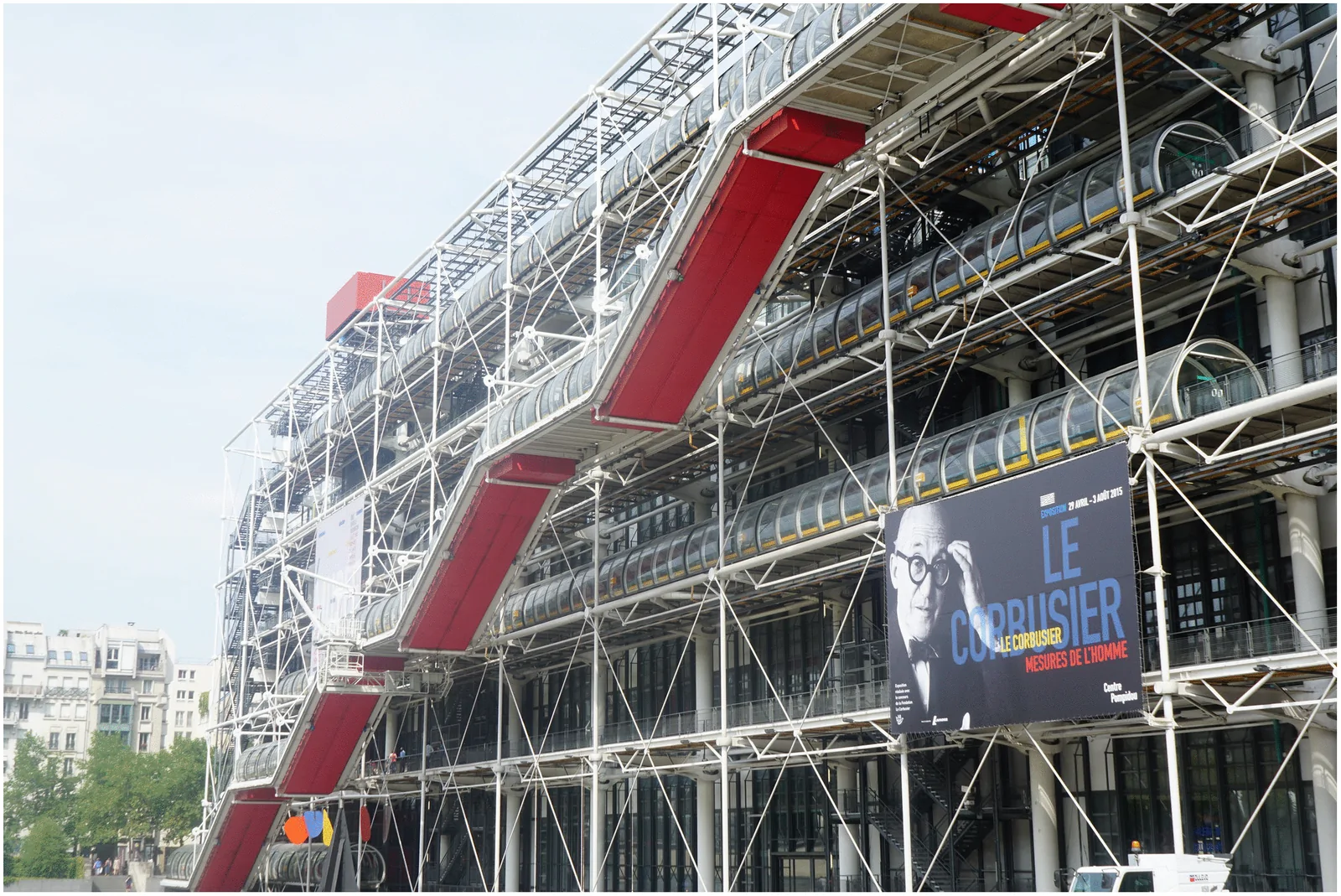
Centre Pompidou, Paris, France, designed by Renzo Piano and Richard Rogers. Image via Flickr, public domain.

Breakthrough in architecture is reflected in the use of innovative materials and design concepts, as demonstrated by Frank Gehry’s titanium cladding in Walt Disney Concert Hall [27] and the exposed technological systems of the Centre Pompidou, as illustrated in (Figs 11 and 12) [28,29]

**Fig 13:**
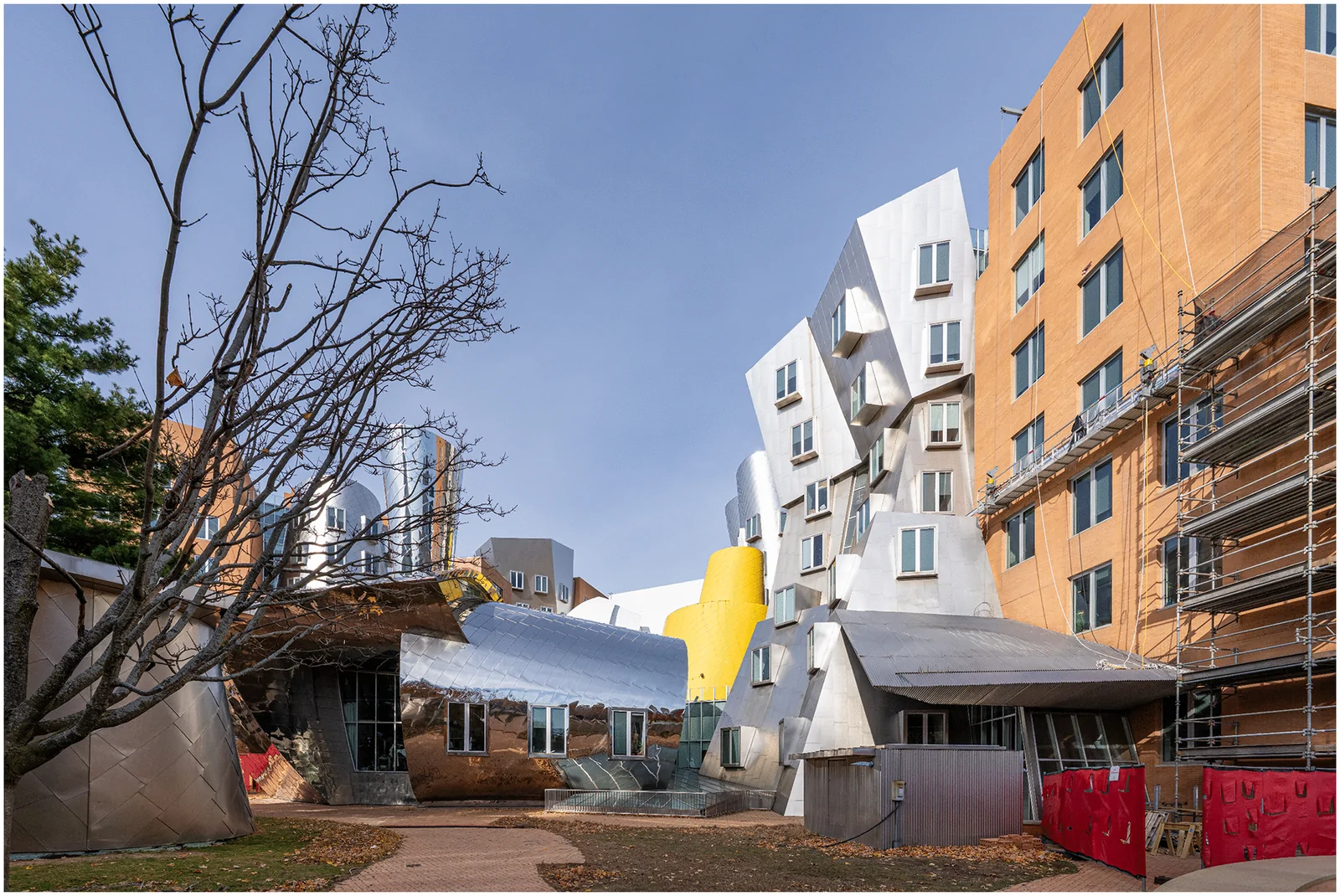
Stata Center, Massachusetts Institute of Technology (MIT), Cambridge, USA. Designd by Frank Gehry. Photograph by ajay_suresh, via Wikimedia Commons (originally from Flickr), licensed under CC BY 4.0.

- **Building of confrontational forms (deconstruction)**: This architectural approach involves deconstructing traditional forms and rearranging them in unconventional ways, emphasizing dynamism and movement. It breaks away from conventional urban and architectural patterns. A prominent example is the Ray and Maria Stata Center at the Massachusetts Institute of Technology (MIT), designed by Frank Gehry in 2004, as illustrated in (Fig 13). Its unconventional, fragmented form reflects intellectual dynamism and supports creativity and innovation within multidisciplinary scientific environments. [30].

### Analytical framework: Indicators for assessing architectural breakthroughs

Based on the preceding theoretical framework, the concept of architectural breakthrough is operationalized into a structured set of measurable indicators to enable systematic analysis of the selected case studies. The extracted measures consist of four main indicators, each supported by secondary sub-indicators, derived directly from previous theoretical and empirical studies. These indicators constitute the final analytical framework adopted in this study and are applied in the subsequent methodological and analytical phases.

To clarify the conceptual structure of the study, architectural breakthroughs are organized into three analytical layers. First, the theoretical dimensions (intellectual, cultural, cognitive, and social) define the broad domains through which breakthrough operates conceptually. Second, the levels of breakthrough (intellectual, productive, material, and experimental) describe how these dimensions manifest within architectural production. Third, the indicators of breakthrough (design innovation, sustainability and technological integration, interaction with local context, and functional and spatial quality) operationalize the previous two layers into measurable criteria applied in the empirical case study analysis.

Accordingly, Table 1 summarizes the theoretical synthesis of dimensions and levels, while Table 2 represents the practical application of the derived indicators in evaluating the case studies.

**Table 1:** Analytical Framework: Indicators and Sub-indicators for Assessing Architectural Breakthrough.

Main Indicator	Definition/ Analytical Focus	Secondary Sub-indicators (Measurement Dimensions)	Key Theoretical Sources
**1. Design Innovation**	Evaluates the degree to which the project departs from conventional architectural norms and paradigms.	• Formal novelty & non-traditional geometry • Experimental structural systems & techniques • Rejection or radical reinterpretation of established compositional rules	Tschumi, 1996 [13]; Gehry (via Stenberg, 2009) [30]; Deconstructivist principles [23]
**2. Environmental Sustainability & Technological Integration**	Assesses the integration of ecological responsibility and advanced technologies into design and construction.	• Use of sustainable or energy-producing materials • Implementation of energy-efficient environmental systems (lighting, HVAC) • Adoption of innovative construction technologies & smart systems	Al-Assadi et al., 2021 [25,26]; High-Tech/Sustainable design [28,29]; BREEAM/LEED principles
**3. Interaction with Local Context & Cultural Identity**	Measures how architecture negotiates its relationship with the local cultural heritage and socio-urban fabric.	• Integration of cultural/heritage symbols with contemporary interpretation • Use of local or context-sensitive materials and motifs • Responsiveness to the social character and urban morphology of the surroundings	Bullock, 2009 [8]; Bassim & Alobaydi [4]; Identity theories [1,14]
**4. Functional & Spatial Quality**	Evaluates the effectiveness of the building in fulfilling its practical objectives and enhancing human experience.	• Spatial efficiency and clarity of user circulation • Flexibility and adaptability of spaces for varied uses • Contribution to user comfort, well-being, and quality of life	Drexler, 1979 [7]; Modernist function-form principles [17]; User-centered design

This framework provides the structured lens for the subsequent systematic application and comparative analysis in the case studies.

**Table 2:** Operationalization of Theoretical Dimensions into Analytical Indicators for Assessing Architectural Breakthrough.

Theoretical Dimension	Corresponding Analytical Indicator	Operationalization Mechanism (How Theory Becomes Measurable)
Intellectual	Design Innovation	The intellectual dimension relates to introducing new critical and philosophical concepts that challenge established knowledge in design. It was operationalized into an indicator that measures formal novelty, experimental structural systems, and radical reinterpretation of traditional compositional rules. The more daring a project is in its form and experimentation in its structure, the more it expresses the intellectual dimension of breakthrough.
Cognitive	Environmental Sustainability & Technological Integration	The cognitive dimension relates to expanding perceptual horizons and reinterpreting the relationships between matter, space, and meaning. It was operationalized into an indicator that measures the use of new materials, energy-efficient systems, and innovative construction technologies that expand the boundaries of architectural knowledge. The more advanced a project is in its use of modern technologies and materials, the more it expresses the cognitive dimension of breakthrough.
Cultural	Interaction with Local Context & Cultural Identity	The cultural dimension relates to the impact of cultural elements on society, its values, customs, and traditions. It was operationalized into an indicator that measures the integration of heritage symbols, the use of context-sensitive materials, and responsiveness to the social and urban character of the place. The more a project connects with its local heritage and cultural identity, the more it expresses the cultural dimension of breakthrough.
Social	Functional & Spatial Quality	The social dimension relates to changing the nature of interaction between humans and public space or the built environment. It was operationalized into an indicator that measures spatial efficiency, flexibility and adaptability of spaces, and their contribution to user comfort, well-being, and quality of life. The more effective a project is in meeting user needs and improving their experience, the more it expresses the social dimension of breakthrough.

**Fig 14:**
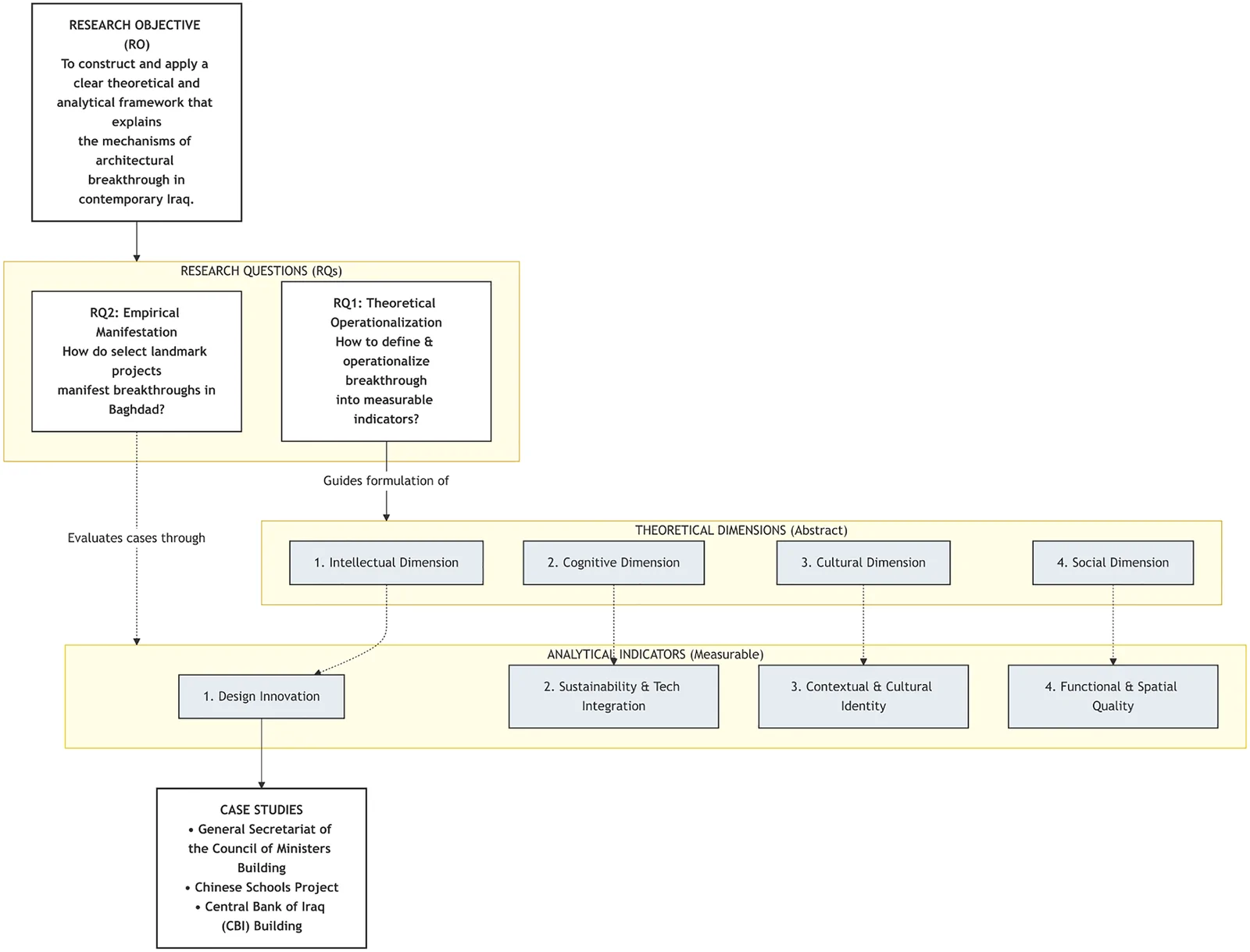
Methodological Operationalization and Linkage Model between Research Questions, Theoretical Dimension and Analytical Indicators (created by author).

To create a strong methodological connection, the four theoretical dimensions and the respective indicators are introduced along with the core research questions (RQs) and explicitly link each indicator to the corresponding research question (Fig 14) illustrates. The Intellectual dimension maps onto Design Innovation (where the formal and geometric novelty are assessed); the Cognitive dimension is operationalized through Environmental Sustainability & Technological Integration (where the expansion of building knowledge is assessed); the Cultural dimension is translated into Interaction with Local Context & Cultural Identity (where the interaction between user and building is assessed); and the Social dimension is operationalized through Functional & Spatial Quality (where the building’s user-space dynamics are assessed).

#### Summary of analytical indicators.

Given the above theoretical analysis, the notion of a breakthrough in architecture is operationalized in four analytical signposts that will be systematically explored with regard to the selected case studies:

1. Design Innovation: assesses how far the project is from standard architectural practices, both formally, by being novel, and structurally, by being experimental, and by radical reinterpretation of known architectural compositional rules.
2. Environmental Sustainability and Technological Integration: Evaluate integration of ecological responsibility by sustainable materials, energy-efficient systems and the use of innovative construction technology.
3. Interactions with Local Context and Cultural Identity: The way an architecture relates to the local cultural context such as the incorporation of cultural references, selection of appropriate materials and sensitivity towards social and urban context.
4. Spatial Quality and Functional Quality: Assesses the suitability of the building for carrying out its functional purpose such as spatial efficiency and flexibility of spaces and its contribution for user comfort and well-being.

The Research Methodology section will use these four indicators as a structured analytical lens for the comparative case study analysis, which is derived from the theoretical synthesis presented above and summarized in Table 1. The three case studies are comparable and use the same indicators for each of the indicators to ensure methodological coherence.

#### Reconciliation of theoretical dimensions and analytical indicators.

Based on the preceding theoretical discussion, four theoretical dimensions of architectural breakthrough were identified: the cultural dimension, the intellectual dimension, the cognitive dimension, and the social dimension. However, these theoretical dimensions represent abstract concepts that cannot be measured directly; therefore, they were transformed (operationalized) into four measurable analytical indicators, as presented in Table 2. The following table illustrates the mapping between these dimensions and indicators, along with an explanation of the operationalization mechanism for each:

#### Synthesis of methodological operationalization:.

The four analytical indicators—Design Innovation, Environmental Sustainability and Technological Integration, Interaction with Local Context and Cultural Identity, and Functional and Spatial Quality—represent the systematic methodological operationalization of the four theoretical dimensions—Intellectual, Cognitive, Cultural, and Social—respectively. Each indicator was designed to measure the physical and architectural manifestation of its corresponding theoretical dimension, enabling an objective and measurable assessment of architectural breakthrough in the case studies.

Thus, the analytical framework bridges theory and practice by transforming abstract concepts (theoretical dimensions) into tangible criteria (analytical indicators) that can be observed and evaluated in actual architectural projects. This methodological transformation ensures that the analysis is not merely a superficial description, but is grounded in a robust theoretical foundation that enables a deep understanding of how architectural breakthrough is realized in the contemporary Iraqi context.

## Research methodology

This study adopts a qualitative research approach to investigate the concept of architectural breakthrough through a comparative case study analysis. The research is structured around a systematic methodological framework that integrates multiple sources of evidence to ensure analytical rigor, validity, and consistency. A triangulation strategy was employed, combining field observation, document analysis, and semi-structured interviews to provide a comprehensive understanding of the selected cases.

Data was analyzed using reflexive thematic analysis supported by NVivo software, while an evidence-based scoring framework was applied to evaluate performance across four key indicators. The following subsections provide a detailed explanation of each methodological component.

### Research design

The study utilizes a multiple case study design focusing on three contemporary architectural projects in post-2003 Iraq. The selected cases represent different architectural typologies and institutional contexts, allowing for a comparative investigation of how architectural breakthrough manifests across varying conditions. A unified analytical framework, derived from the Theoretical framework section, was applied consistently across all cases to ensure methodological coherence and comparability.

1 **The General Secretariat of the Council of Ministers Building:**

This project represents a major governmental complex in Baghdad designed to accommodate the administrative headquarters of the Iraqi Council of Ministers. The building was conceived as a contemporary architectural landmark reflecting the institutional authority of the Iraqi state while expressing a modern architectural identity. Its design emphasizes symbolic presence, formal innovation, and spatial organization that supports administrative functions, while simultaneously projecting an image of governmental transparency and modernity within the urban context.

2 **The Chinese Schools Project:**

The selection of the Chinese Schools project was intentional, as it represents a different typology and scale compared to the landmark state-driven projects. The aim was not to compare projects based on formal monumentality, but to examine how breakthrough manifests across different architectural contexts and programmatic conditions. Including a large-scale public typology allows the study to test whether breakthrough is limited to symbolic projects or can also emerge through systemic functional and spatial innovation.

3 **The Central Bank of Iraq Building:**

Represents a high-profile financial institution project prioritizing technological integration and sustainability. These cases were selected for their prominence, typological diversity (governmental, educational, financial), and explicit aspirations toward architectural distinction in the post-2003 Iraqi context, making them rich sites for investigating the phenomenon of breakthrough which will be described in detail.

### Data collection methods

Data was collected from three primary sources to enable triangulation:

- **Field Observation and Visual Documentation:**

Field observations and photographic documentation were conducted in publicly accessible urban locations in Iraq. The architectural sites included in the study are public buildings and landmarks that can be freely observed and photographed from public spaces. In the Iraqi context, no formal permits are required for observing or photographing publicly accessible buildings. Therefore, no formal fieldwork permits were required for this study.

- **Document and Archival Analysis:**

A range of project-related materials was reviewed, including architectural drawings (plans, sections), renderings, official project descriptions, consultant reports, and relevant media sources. These documents were analyzed to understand design intent, technical characteristics, spatial organization, and the conceptual narrative underlying each project. Architectural plans were specifically analyzed to examine spatial organization, functional zoning, and circulation efficiency, particularly in relation to the indicators of functional and spatial quality and design innovation. Particular attention was given to architectural plans to examine functional distribution, circulation efficiency, and spatial relationships in relation to breakthrough indicators.

- **Semi-Structured Interviews:** In-depth semi-structured interviews were conducted with key stakeholders, including architects, academics, planners, and users. The interview questions were structured around the main analytical indicators of the study—design innovation, sustainability and technological integration, contextual interaction, and functional and spatial quality. Participants were asked to evaluate the extent of innovation, identify key design features, and reflect on the project’s contribution to architectural transformation. Field notes were recorded during and after interviews and site visits to capture additional contextual and observational insights.

The sample comprised:

- Four (4) practicing architects involved in contemporary Iraqi projects.
- Three (3) academic researchers specializing in architectural theory, history, or criticism related to Iraq/the region.
- Two (2) urban designers/planners familiar with Baghdad’s urban development.
- One (1) facility/user representative with direct experience of one of the case study buildings.

The ten participants were selected via purposive sampling to ensure representation of relevant expertise. They were approached via direct professional contact and invited to participate through phone contact or online communication prior to scheduling the interviews, no repeat interviews were conducted. Each participant was interviewed once. Each interview lasted between 20–25 minutes and was conducted online (via video communication), based on participant availability. A semi-structured interview guide was used, with open-ended questions aligned with the four analytical indicators: Design Innovation, Sustainability and Technological Integration, Contextual Interaction and Cultural Identity, and Functional and Spatial Quality.

All interviews were audio-recorded with informed verbal consent, transcribed verbatim, and anonymized (participants were assigned coded identifiers such as P1, P2).

The interview protocol was explicitly aligned with the analytical framework, ensuring that each question corresponded to one or more indicators. Participants were asked to evaluate the degree of breakthrough, identify key architectural features, and reflect on the projects’ contribution to architectural transformation. Example questions included: “How does this project depart from conventional architectural approaches?” and “In what ways does the design demonstrate innovation in spatial organization or user experience?”

The characteristics of the participants are summarized in Table 3.

**Table 3:** Profile of Interview Participants.

Participant Code	Professional background	Years of Experience	Interview Mode	Duration (Minutes)
**P1**	Architect (Design Practice)	15+	Online	25
**P2**	Architect (Design Practice)	10-15	Online	25
**P3**	Architect (Design Practice)	12	Online	20
**P4**	Architect (Design Practice)	7	Online	25
**P5**	Academic (Architecture)	20+	Online	25
**P6**	Academic (Architecture)	18	Online	20
**P7**	Academic (Architecture)	14	Online	25
**P8**	Urban Planner/ Designer	11	Online	20
**P9**	Urban Planner/ Designer	13	Online	25
**P10**	Facilities Manager/ User Rep.	8	Online	25

### Qualitative data analysis

The interview transcripts, along with field notes, were analyzed using Reflexive Thematic Analysis (TA) following the six-phase framework established by Braun and Clarke [31]. The analysis was supported by NVivo 14 software for data management, coding, and theme development. Thematic sufficiency was achieved during data collection. Interview transcripts were not returned to participants for review or correction. This was because re-contacting all participants after transcription was not logistically feasible due to scheduling constraints, limited participant availability, and the practical difficulty of arranging follow-up communication. Nevertheless, the accuracy and trustworthiness of the data were ensured through verbatim transcription, cross-checking against the original audio recordings, and systematic verification before analysis.

**Familiarization:** The researchers immersed themselves in the data by repeatedly reading transcripts and listening to audio recordings.

**Initial Coding:** Systematic, data-driven coding was performed line-by-line to identify features of potential analytical interest. Codes were generated inductively but remained sensitized by the four core indicators from the theoretical framework.

**Theme generation:** Codes were collated and examined for patterns of shared meaning. Candidate themes were constructed by grouping related codes into broader, cohesive ideas that captured something significant for architectural breakthroughs in the data.

**Theme Review:** Candidate themes were reviewed and refined at two levels: first by checking the coded extracts within each theme for coherence, and second by considering the validity of the entire thematic map in relation to the full dataset. This iterative process involved merging, splitting, or discarding themes.

**Theme Definition and Naming:** Each finalized theme was clearly defined and given a concise, informative name that captured its essence. The scope and boundaries of each theme were articulated.

**Report Production:** The analysis was woven into a coherent narrative for the Results section, supported by vivid, illustrative data extracts (quotations) from the participants.

**Adherence to qualitative research reporting guidelines**

This study follows established qualitative research reporting standards. The design, data collection, and analysis procedures were guided by the Consolidated Criteria for Reporting Qualitative Research (COREQ), ensuring transparency in sampling, data collection, coding, and interpretation processes. A complete COREQ checklist is provided as Supplementary Material (S1 File).

### Validation and analytical rigor (Trustworthiness)

To ensure the credibility and trustworthiness of the qualitative findings, multiple strategies were employed:

**Analyst Triangulation:** Both authors independently coded a subset of three interview transcripts and then collaboratively discussed and reconciled the coding schemes and emerging themes.

**Data Triangulation:** Findings from the interviews were systematically cross-referenced and compared with evidence from field observations and document analysis for convergence or divergence.

**Thick Description:** Detailed, contextual descriptions of the case studies and participant quotes are provided to allow readers to assess the transferability of findings.

**Reflexivity:** The researchers’ positions as academics in Iraqi architecture are acknowledged, and efforts were made to balance insider understanding with critical analytical distance. The researcher is trained in qualitative interviewing and has prior experience in conducting semi-structured interviews and thematic analysis. No prior relationship was established between the researcher and the participants before the commencement of the study.

### Quantitative scoring framework for case study comparison

To facilitate a structured comparative analysis, a 5-point ordinal scoring scale (1–5) was applied to each case study for the four main indicators. The numerical scores were derived systematically through triangulation of evidence obtained from field observations, document analysis, and interview data. Each indicator score was determined by evaluating the consistency and strength of evidence across the three data sources according to the predefined scoring rubric presented in Table 4. A score of 5 (Exceptional) required strong, consistent evidence from all three sources, while a score of 1 (Absent) indicated no supporting evidence from any source. The final score for each project represents a consensus judgment based on this evidence-based rubric, and the overall project score presented in the results corresponds to the arithmetic mean of the four indicator scores.

**Table 4:** Evidence-Based Scoring Rubric for Quantitative Assessment of Architectural Breakthrough.

Score	Label	Definition & Evidence Requirements for Each Score
**5**	Exceptional	Strong, consistent, and corroborating evidence from all three data sources (Triangulation Achieved). Field observation confirms outstanding execution and impact; documents provide clear proof of innovative intent/performance; interviews reveal expert consensus on the project’s breakthrough status for the specific indicator.
**4**	Very Good/ Good	Substantial evidence from at least two sources, with no contradictory evidence from the third. Design meets or exceeds key objectives with minor gaps. Evidence is generally positive and aligned, though not uniformly exceptional across all sources.
**3**	Adequate/ Moderate	Mixed or moderate evidence. Implementation is present but basic, lacking sophistication or consistency. Evidence may be positive in one source (e.g., documents claim innovation) but neutral or weakly supportive in others (e.g., field observation shows a standard execution).
**2**	Limited/ Weak	Evidence is weak, limited, or contradictory. Only one source provides clear support, or sources contradict each other on key aspects. Implementation is partial, inconsistent, or fundamentally problematic.
**1**	Absent/ Very Poor	No supporting evidence from any source, OR all sources confirm failure/absence. The indicator is not addressed, or the attempt is fundamentally flawed/non-functional.

**Validation Note:** Two researchers independently applied this rubric. Inter-rater reliability was high (Cohen’s Kappa > 0.75). Discrepancies were resolved through discussion with reference to the triangulated evidence.

#### Ethics statement.

All participants involved in this study were professional architects, including both academic researchers and practicing architects. Prior to the interviews, participants were fully informed about the purpose of the research, the voluntary nature of their participation, and their right to withdraw from the study at any stage without any consequences. Verbal informed consent was obtained from all participants before the interviews were conducted. The consent process was documented by the researchers in the interview records. No minors or vulnerable individuals were involved in this research.

## Results

All case studies were analyzed using a unified set of four predefined analytical indicators—**Design Innovation, Sustainability and Technological Integration, Contextual Interaction and Cultural Identity, and Functional and Spatial Quality**—to ensure methodological consistency and comparability across the selected projects. The results presented in this section are derived from a triangulated analytical framework combining field observations, architectural documentation, design drawings, and semi-structured interviews with practicing architects.

The presentation of results proceeds from an analytical examination of each indicator across the case studies to a consolidated interpretation of the findings. Qualitative evidence obtained from interviews with professional architects was used to contextualize and interpret the observed architectural characteristics, providing insight into how practitioners perceive contemporary transformations in Iraqi architectural practice.

1 Design innovation

The analysis indicates that the selected projects demonstrate varying degrees of formal and conceptual innovation. Several case studies reveal the adoption of contemporary design strategies that reinterpret traditional spatial principles within modern architectural language. This innovation is reflected in the manipulation of form, spatial hierarchy, and material expression.

Interview data supports these observations and highlights how architects perceive innovation in the Iraqi architectural context. As one architect explained:

“Architectural innovation in Iraq today does not necessarily mean abandoning tradition, but rather reinterpreting inherited spatial concepts through contemporary design approaches.”

*(Architect A, interview)*

Another participant emphasized that innovation often emerges through gradual transformation rather than radical departure:

“What we see today is not a complete break from the past, but a gradual evolution where architects reinterpret local architectural values within modern design frameworks.”

*(Architect B, interview)*

These insights suggest that architectural breakthrough in contemporary Iraqi practice often occurs through reinterpretation rather than rejection of tradition.

2 Sustainability and technological integration

The analysis of the selected projects reveals increasing attention to environmental considerations and the integration of modern building technologies. Several case studies demonstrate attempts to incorporate passive environmental strategies, material efficiency, and technological systems aimed at improving building performance.

Architects interviewed in this study emphasized the growing importance of sustainability within contemporary design practice. As one participant noted:

“Sustainability is becoming an essential design consideration in contemporary Iraqi architecture, particularly in response to environmental and climatic challenges.”

*(Architect C, interview)*

Another architect highlighted the role of technological integration in enabling innovative architectural solutions:

“New technologies allow architects to experiment with forms and materials while also improving environmental performance.”

*(Architect D, interview)*

These findings suggest that sustainability and technology are increasingly shaping architectural innovation in the Iraqi context.

3 Contextual interaction and cultural identity

The results indicate that many contemporary projects attempt to establish a meaningful relationship with their surrounding urban and cultural context. Several buildings demonstrate efforts to integrate elements of local identity, whether through spatial organization, material selection, or symbolic references.

Interview participants frequently emphasized the importance of maintaining cultural continuity within contemporary architectural production. As one architect stated:

“The challenge for Iraqi architects today is to innovate while preserving the cultural depth of our architectural heritage.”

*(Architect E, interview)*

Another architect described contextual interaction as a key dimension of contemporary design:

“Architecture should respond to its place—its climate, culture, and urban environment—otherwise it loses its connection with society.”

*(Architect A, interview)*

These observations highlight the continuing relevance of cultural identity in shaping contemporary Iraqi architectural practice.

4 Functional and spatial quality

The evaluation of architectural drawings and spatial configurations shows that functional clarity and spatial efficiency remain central design priorities across the analyzed projects. Several case studies demonstrate careful attention to circulation, spatial hierarchy, and user experience.

Interview data further reinforces the importance of functional quality in contemporary architectural design. One architect explained:

“A successful architectural project must achieve a balance between aesthetic expression and functional performance.”

*(Architect B, interview)*

### Another participant emphasized the importance of spatial experience

“Architecture should not only be visually expressive but also provide meaningful spatial experiences for its users.”

*(Architect C, interview)*

These findings indicate that functional and spatial considerations remain fundamental components of architectural quality alongside formal and conceptual innovation.

### General secretariat of the council of ministers

**Fig 15:**
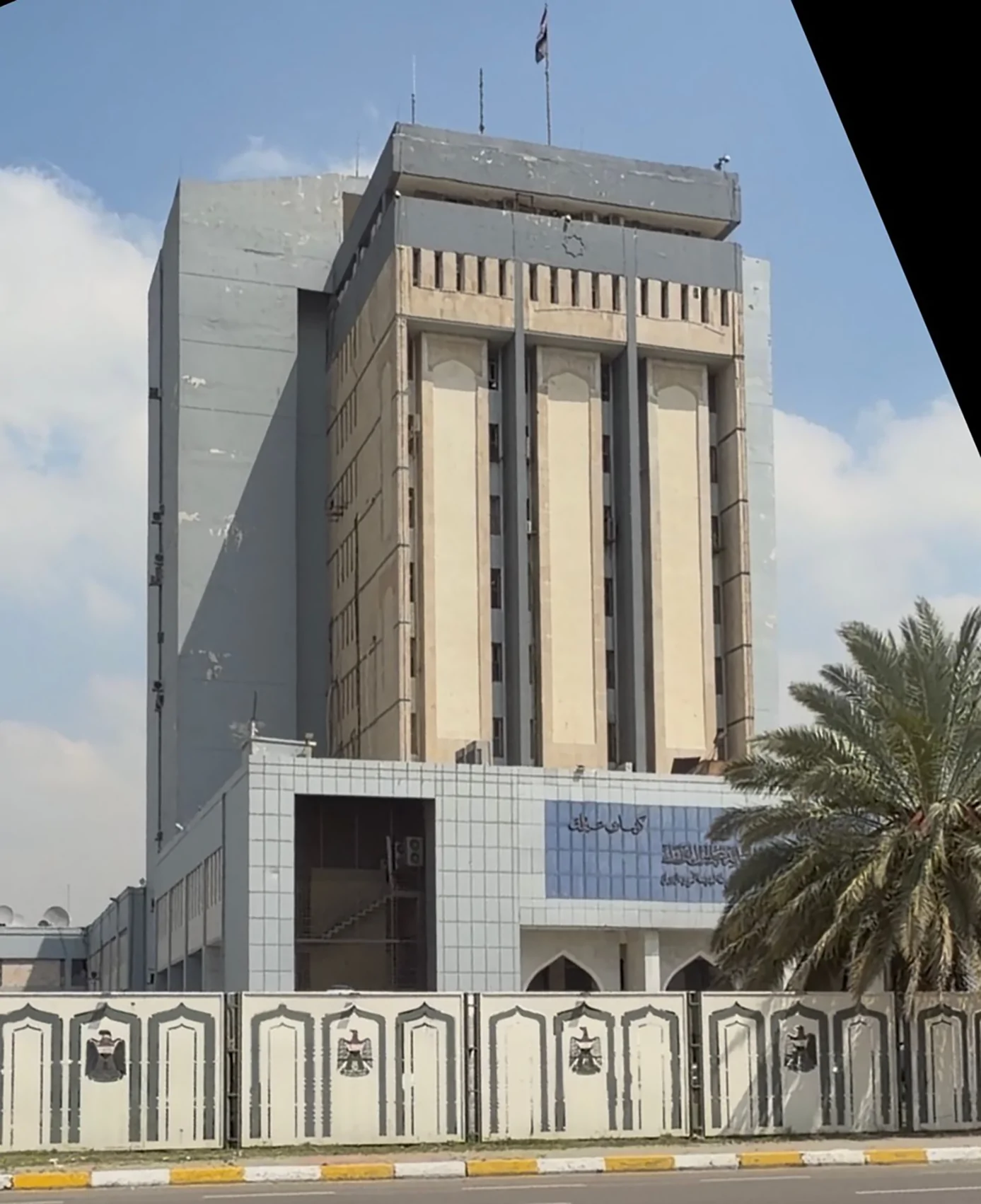
Old Iraqi Council of Ministers building Source (by authors).

**Fig 16:**
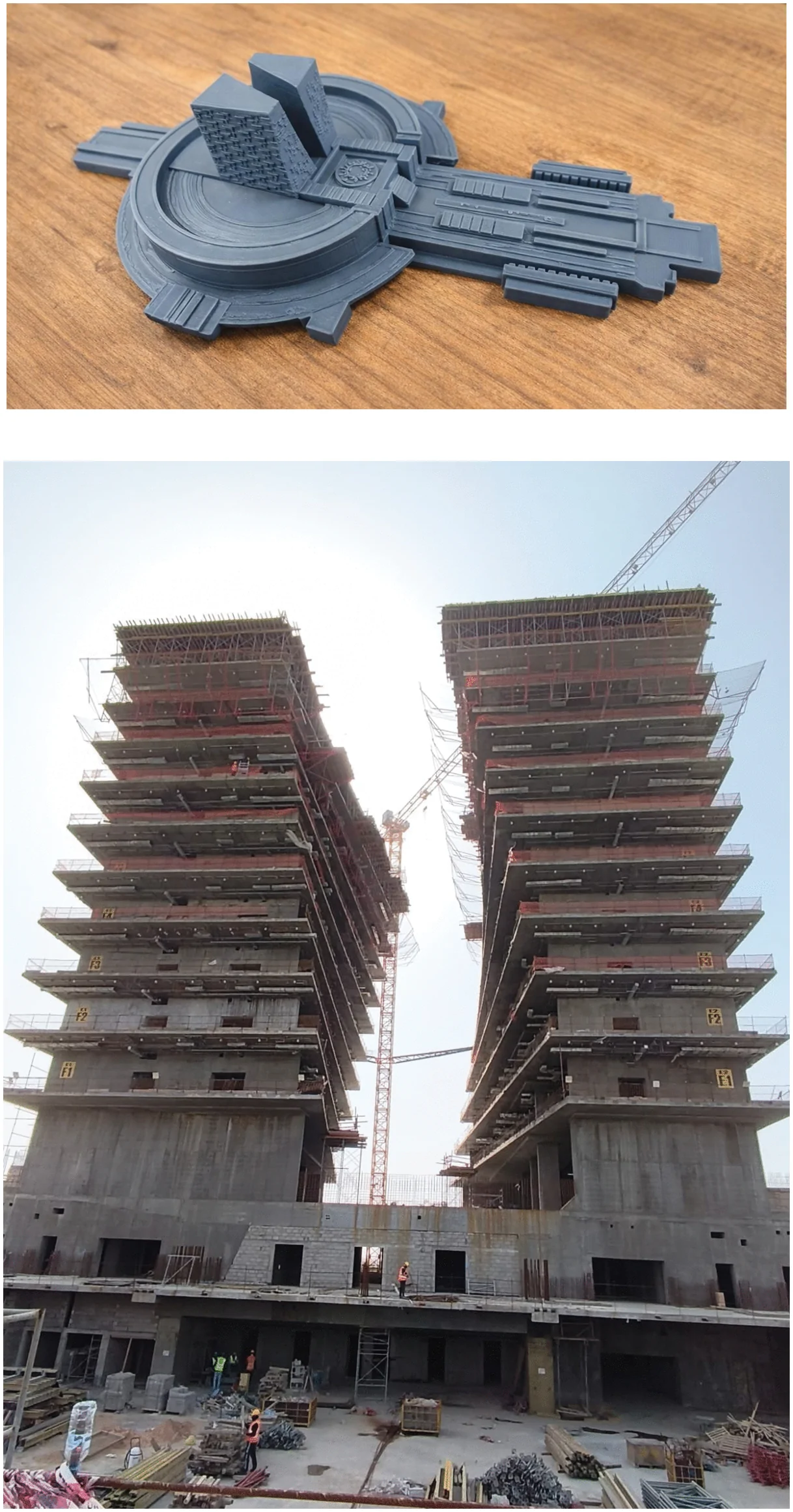
a. Conceptual model of the new Iraqi Council of Ministers building (created by authors). **b. 3D-printed model of the new Iraqi Council of Ministers building, showing the building’s massing and spatial relationships** (created by authors).

A government project designed by architect Manhal Al-Habobi. It includes a 17-story main tower and various administrative facilities, built to expand workspace and enhance institutional capacity. The design follows modern construction approaches, incorporating adaptive systems and energy-efficient lighting, as identified in project documentation. Architecturally, it incorporates references to cuneiform and Islamic motifs through abstract formal composition, with two curving towers emerging from a central volumetric mass. These features are described in previous studies (Bassim & Alobaydi) The transformation from the former governmental architectural language to the contemporary reinterpretation of institutional identity is illustrated through) Figs 15 and 16) [4].

### Design innovation

The General Secretariat building exhibits a distinct architectural composition characterized by dynamic massing and symbolic abstraction. Field observations documented variations in façade geometry and volumetric hierarchy, contributing to its visual prominence within the urban context. Interview participants (e.g., P10, P7) identified the reinterpretation of cultural references as a key design feature (Table 6), particularly in relation to the abstraction of historical motifs into contemporary architectural form, specifically referencing the Iraqi “symbol of eternity,” a two-dimensional motif reinterpreted into a three-dimensional architectural form. This transformation is expressed through the creation of a central void within the building mass, symbolically representing continuity, infinity, and the cultural identity of Mesopotamia, as indicated in architectural descriptions and prior studies (Bassim & Alobaydi) [4].

**Table 6:** Consolidated Quantitative Evaluation of Case Studies Based on Main Indicators.

Analytical Indicator	General Secretariat Building	Chinese Schools Project	Central Bank of Iraq Building	Key Supporting Evidence (Source)
**1.Design Innovation**	5/5	3/5	5/5	Sec./Bank: Non-traditional, symbolic geometry (Field Obs., Docs). Schools: Modern but modular/functional focus (Docs, Field Obs.).
**2. Environmental Sustainability & Tech. Integration**	4/5	4/5	5/5	Bank: BREEAM pursuit (Doc.), advanced systems (Field Obs., Int.). Sec./Schools: Good standards (efficient lighting, glass) but not pioneering (Field Obs., Int.).
**3. Interaction with Context & Cultural Identity**	5/5	4/5	4/5	Sec.: Cuneiform seal as generative concept (Doc., Int.). Schools: Adaptable but generic modern aesthetic (Int., Field Obs.). Bank: Modern symbolism over heritage dialogue (Int., Field Obs.).
**4. Functional & Spatial Quality**	4/5	5/5	4/5	Schools: Optimal lighting, color psychology, open plans (Field Obs., Int.). Sec./Bank: Grand symbolic spaces; some critiques of daily-user circulation (Int.).
**Overall Average Score**	4.5	4.0	4.5	

### Sustainability and technological integration

The project incorporates advanced structural systems and contemporary construction technologies. However, environmental sustainability strategies appear to be secondary compared to formal expression. Document analysis and expert interviews suggest that technological integration primarily supports structural ambition rather than passive environmental optimization.

### Contextual interaction and cultural identity

The building engages deeply with Iraqi cultural identity through conceptual reinterpretation rather than literal replication. Participants consistently highlighted the intellectual embedding of heritage motifs as a defining characteristic of breakthrough (Table 6). The project contributes symbolically to redefining national architectural identity. It expresses references to local architectural identity primarily through formal and symbolic articulation rather than through explicitly documented use of local construction materials.

### Functional and spatial quality

Architectural plans indicate clear zoning and hierarchical circulation. Field observations confirmed effective spatial organization and user movement patterns within administrative areas. However, functional innovation remains aligned with conventional governmental programming rather than radical reconfiguration.

### Chinese schools project

represents a large-scale governmental initiative aimed at expanding educational infrastructure in Iraq. Based on standardized design models with varying capacities (e.g., 12, 18, and 24 classrooms), the project introduces a programmatic and typological shift in school design. Field observations documented spatial organization and circulation patterns, particularly in high-usage areas, supporting the assessment of functional and spatial performance. Interview responses (e.g., P5, P8) also referred to the reconfiguration of school layouts compared to conventional Iraqi models (Table 6).

**Fig 17:**
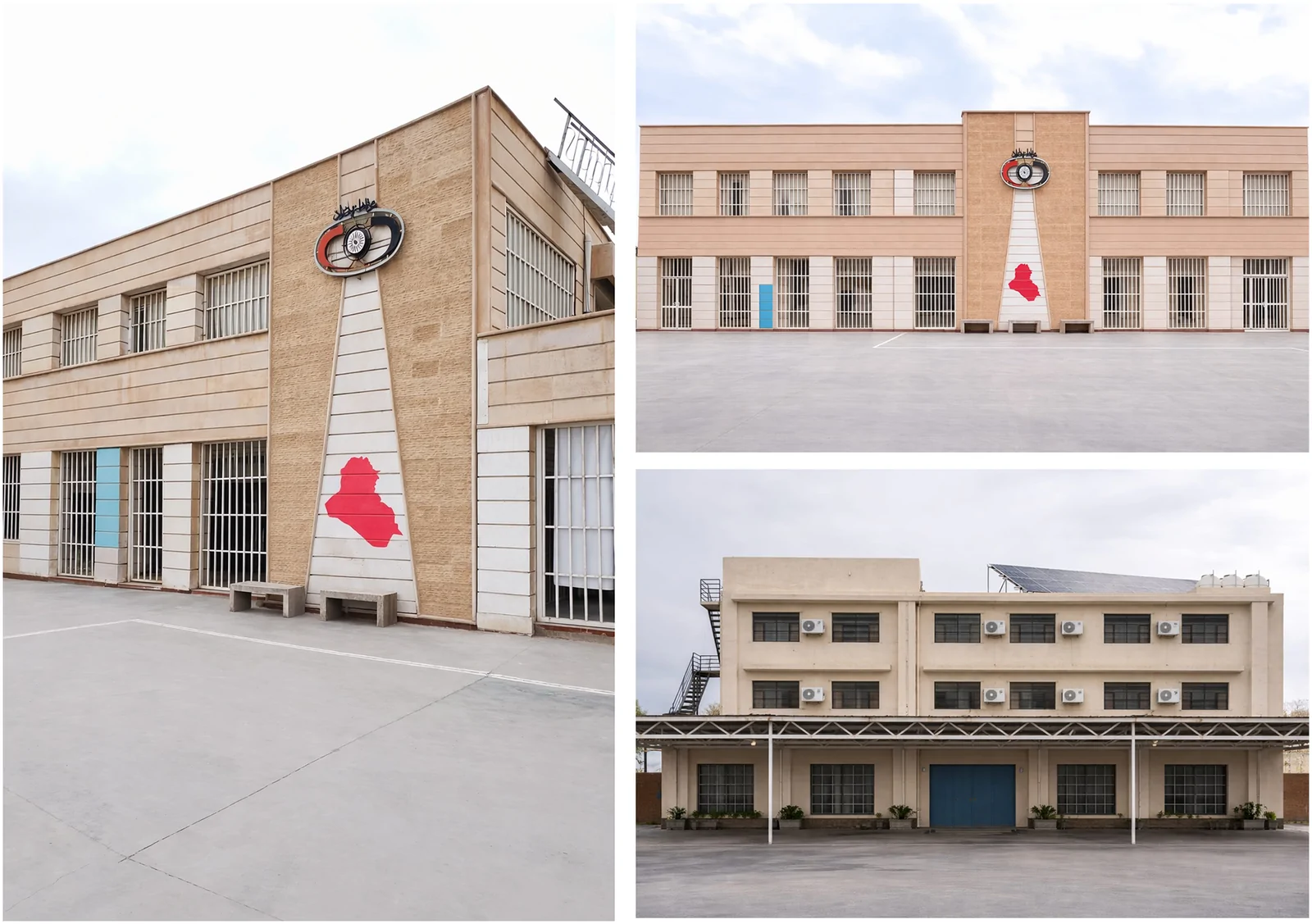
Prevailing school in Iraq (A,B,C create by Authers).

**Fig 18:**
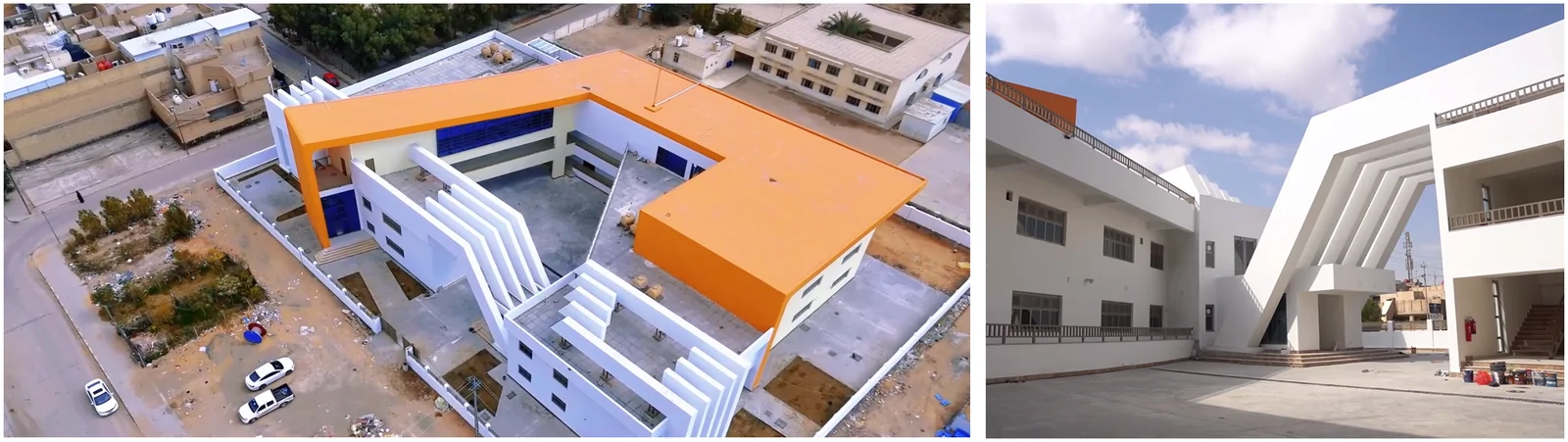
Models of Chinese school (A,B create by Authers).

Geometric configurations and engineering solutions are addressed within the Design Innovation indicator, while aspects related to user experience, openness, and environmental performance are evaluated under the Functional and Spatial Quality and Sustainability indicators. These descriptions are therefore treated as supporting observations within the unified analytical framework rather than as independent categories. To evaluate the extent of the typological and programmatic transformation introduced by the Chinese Schools Project, the prevailing Iraqi school models were systematically compared with the new standardized prototypes, as illustrated in (Figs 17 and 18) [32].

### Design innovation

Unlike landmark governmental projects, the Chinese Schools project reflects a shift at the typological and programmatic level rather than through iconic formal expression. Evidence from architectural layouts and interview responses (e.g., P5; Table 6) indicates modifications in spatial organization and user-oriented planning compared to conventional corridor-based school models.

The analysis of design models (Fig 17) identifies variations in massing, façade articulation, and spatial configuration. For instance, some models demonstrate articulated façades combining solid and glazed elements, supporting daylight penetration and visual connectivity, while others adopt horizontal layouts with defined entrance zones and semi-open external spaces. These features were observed in architectural drawings and visual documentation, indicating an emphasis on spatial openness and user interaction.

In addition, repetitive modular systems and large window openings were observed in several models, contributing to daylight access and spatial clarity within classrooms. However, no direct empirical evidence (from documents or interviews) confirms that individual formal features (such as color or entrance size) alone constitute innovation. Therefore, these characteristics are interpreted as supporting elements within broader spatial and typological reconfiguration.

Interview data (e.g., P5) support this interpretation, emphasizing that the primary transformation lies in the reorganization of the educational environment rather than in isolated formal expressions. As noted by one participant, “The project’s real innovation is not in a singular iconic form, but in rethinking the spatial experience of the school environment” (P5).

Breakdown of Design Models and Innovations:

- **Model A (24-classroom capacity):**
  - **Design Concept:** This model features a bold, monolithic form with a distinctive orange façade, creating a strong visual identity. The design employs a largely solid massing punctuated by a prominent, welcoming entrance portal.
  - **Innovation & Breakthrough Aspect:** The use of a vibrant, non-institutional color palette represents a significant shift, aiming to create a stimulating and psychologically positive environment for students. It challenges the somber aesthetic traditionally associated with public educational buildings in Iraq.
- **Model B (24-classroom capacity):**
  - **Design Concept:** This variant presents a more articulated façade, balancing solid stone elements with extensive glazing. The composition suggests a blend of permanence and transparency.
  - **Innovation & Breakthrough Aspect:** The design strategically negotiates privacy and openness. The glass elements promote visual connectivity and daylight, while the stone portions provide enclosure and thermal mass. This approach fosters an inclusive learning environment that supports both focused study and interactive activities.
- **Model C (12-classroom capacity):**
  - **Design Concept:** Characterized by a low-rise, horizontal layout with a wide, canopy-defined entrance and an integrated front yard or plaza.
  - **Innovation & Breakthrough Aspect:** The emphasis is on creating a communal outdoor space that serves as a transition zone and a setting for social and extracurricular activities. This prioritization of the semi-public realm enhances the school’s role as a community node.
- **Model D (18-classroom capacity):**
  - **Design Concept:** Utilizes a repetitive modular bay system with an emphasis on large, ribbon-style windows and fully glazed corner elements to maximize daylight penetration into all classrooms.
  - **Innovation & Breakthrough Aspect:** The primary breakthrough is biophilic and environmental, focusing on student well-being through abundant natural light. The repetitive yet carefully detailed façade creates a rhythm that is both efficient to build and aesthetically coherent.

**Overall Impact:** Collectively, these designs signify a decisive move away from the corridor-centric, inward-looking school model. The breakthrough is predominantly socio-functional and experiential, prioritizing student well-being, community interaction, and a contemporary learning atmosphere. As one interviewee noted, *“The project’s real innovation is not in a singular iconic form, but in rethinking the very experience of the school day for thousands of children”* (P5, Academic). Each school is conceived not just as a facility, but as a “cultural front,” expressing a national aspiration for educational modernization aligned with international standards of pedagogical design.

### Sustainability and technological integration

The project utilizes modular construction systems and repetitive structural elements, as identified in project documentation and visual analysis. This approach supports scalability and rapid implementation across multiple sites. Field observations and document analysis (Fig 14; Table 6) indicate that design strategies emphasize spatial efficiency, daylight access through window openings, and simplified construction systems.

However, available evidence does not indicate the use of clearly defined advanced environmental technologies (e.g., smart systems or specialized materials). Sustainability is therefore reflected primarily in functional efficiency, standardization, and resource optimization rather than explicitly documented passive or active environmental systems. No direct evidence was found confirming the use of specific sustainable materials or advanced façade technologies within the analyzed models.

### Contextual interaction and cultural identity

Contextual engagement is observed primarily at the operational level, responding to the demand for educational infrastructure across different regions. Interview responses (e.g., P6, P8; Table 6) indicate that the project’s impact is associated with its large-scale implementation and its role in improving access to educational facilities.

No direct architectural evidence was identified indicating the incorporation of explicit cultural symbols or heritage-based design references within the analyzed models. Accordingly, the project’s contextual contribution is interpreted through its functional and societal role rather than through formal or symbolic architectural expression.

### Functional and spatial quality

Architectural plans and field observations document organized classroom layouts, clear circulation systems, and the presence of semi-open or shared spaces in several models. These include entrance forecourts, transitional outdoor areas, and open zones that support social interaction and extracurricular activities.

Observed spatial configurations indicate a shift away from inward-looking, corridor-based layouts toward more open and distributed spatial arrangements. Interview responses (e.g., P3, P5) also indicate improved usability, environmental comfort, and spatial clarity compared to conventional school models (Table 6).

Evidence from observations and user-related feedback suggests that this dimension represents the strongest performance aspect of the project, particularly in relation to spatial organization, user interaction, and the creation of a more engaging learning environment.

### Central bank of Iraq

**Fig 19:**
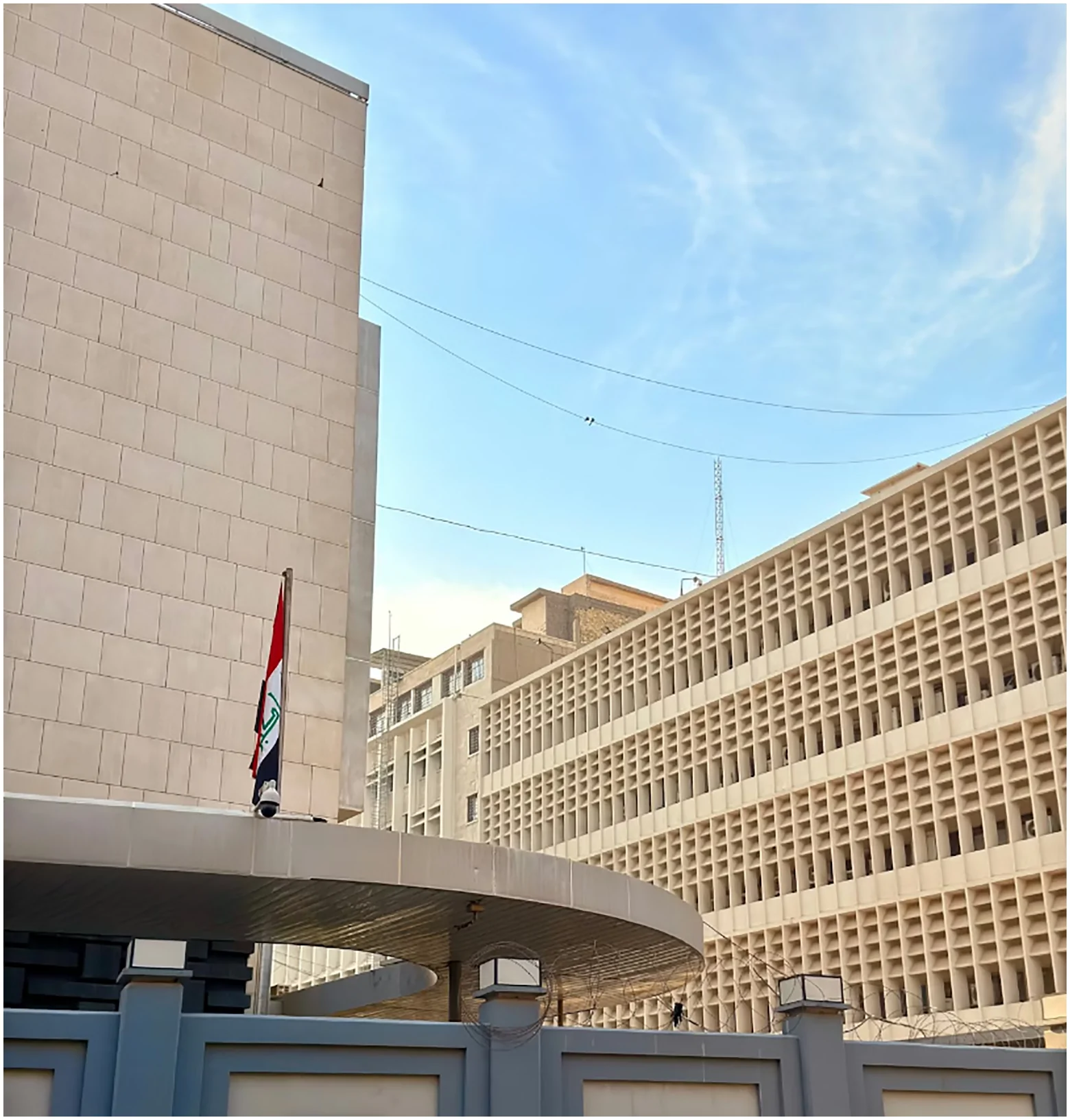
Old central Bank of Iraqi building (Wadaa H., Unsplash (Unsplash License).

**Fig 20:**
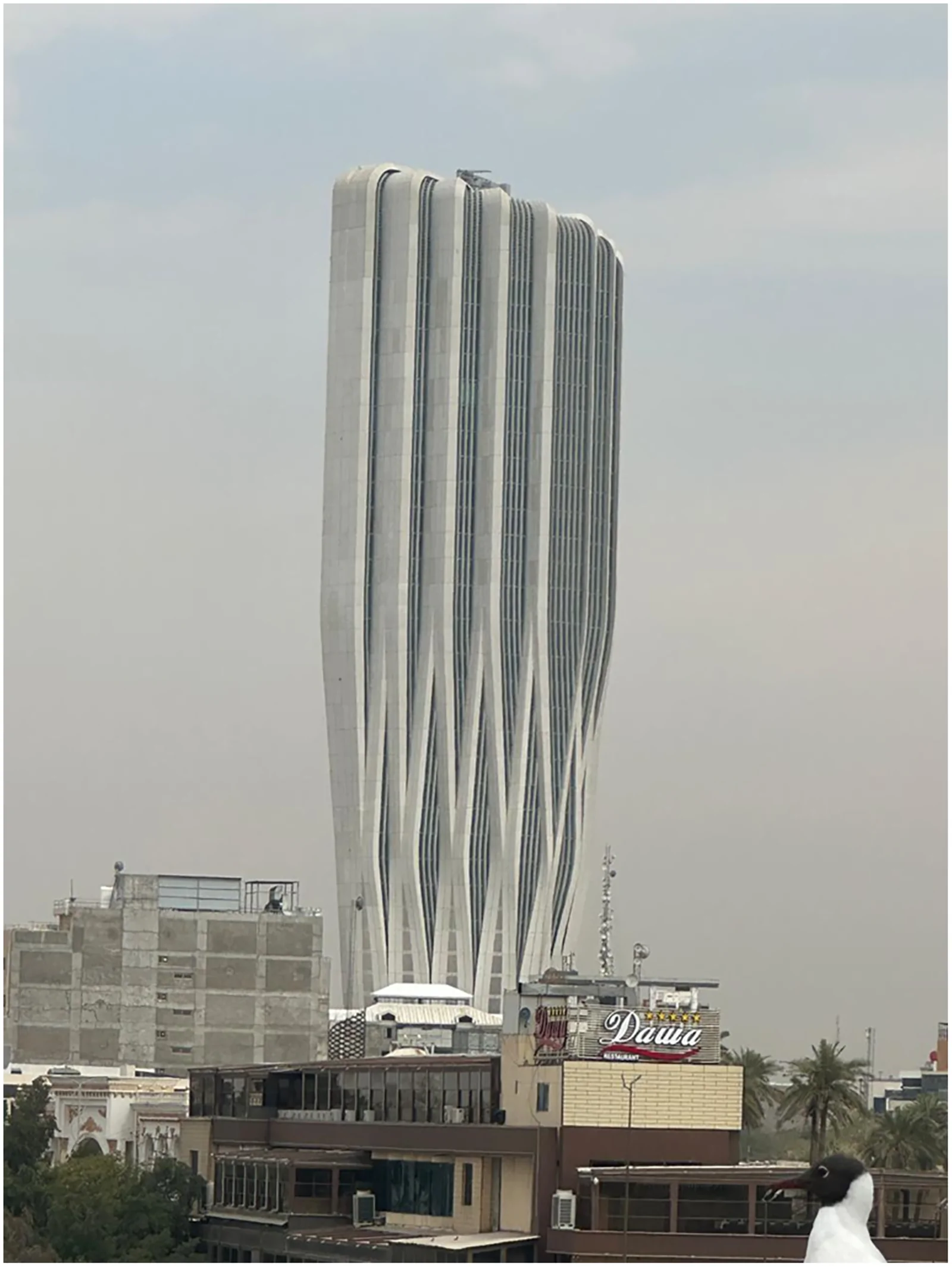
New central Bank of Iraq, Baghdad, Iraq. Designed by Zaha Hadid Architects (created by authors).

The Central Bank of Iraq’s new building is the country’s first to adopt sustainable, environmentally friendly technologies aimed at reducing pollution. Registered for the BREEAM Global Certificate, marks a pioneering step in Iraq and the region toward environmental conservation. The transition from the former institutional banking model to the contemporary sustainable vertical expression is illustrated in (Figs 19 and 20).

### Design innovation

The building exhibits a complex geometric configuration characterized by non-linear forms and layered façade articulation. Visual analysis and project documentation indicate the use of advanced parametric design strategies to generate the building envelope. Interview responses (e.g., P2, P9; Table 6) identify the project’s formal complexity and structural expression as key distinguishing features within the Iraqi architectural context. Field observations document variations in façade depth, surface articulation, and volumetric composition, contributing to the building’s visual prominence within the urban skyline.

### Sustainability and technological integration

Project documentation and secondary sources indicate the use of advanced construction materials and façade systems, including glass-reinforced concrete (GRC) and high-performance glazing systems. These materials support structural performance and façade articulation.

Evidence from façade analysis suggests the presence of layered envelope systems that may contribute to solar control and daylight regulation; however, no detailed publicly available documentation was identified confirming specific environmental technologies such as smart façade systems or dynamic shading devices.

Accordingly, technological integration is primarily evidenced through structural and material systems rather than explicitly documented environmental sustainability strategies.

### Contextual interaction and cultural identity

The building’s formal composition has been interpreted in previous studies as abstractly referencing fluid geometries and contemporary architectural expression rather than explicit historical symbolism. Field observations and visual analysis do not indicate the direct use of identifiable heritage symbols or traditional architectural elements.

Interview responses (e.g., P4, P8; Table 6) suggest that the project’s contribution to identity is associated with its representational role as a national financial institution rather than through direct formal references to Iraqi heritage.

### Functional and spatial quality

Available architectural documentation indicates a hierarchical spatial organization structured around secure zones, administrative areas, and service functions. Circulation systems appear to be organized vertically and horizontally to accommodate operational requirements of a financial institution. Interview responses (e.g., P3, P6) indicate that functional performance aligns with institutional requirements, particularly in terms of security, access control, and spatial hierarchy. No evidence was identified indicating experimental or non-conventional spatial configurations beyond standard high-rise institutional design.

### Comparative analysis of breakthrough signatures

The comparative analysis of the three case studies reveals distinct patterns in the manifestation of architectural breakthrough across the four analytical indicators. Rather than reflecting a uniform model of innovation, the findings demonstrate that breakthrough operates through multiple pathways depending on project type, scale, and institutional context.

The General Secretariat of the Council of Ministers and the Central Bank of Iraq exhibit a consistent pattern of high performance in design innovation and symbolic expression. Both projects demonstrate a strong capacity to redefine architectural identity through formal articulation and conceptual reinterpretation of cultural references. This positions them within a symbolic-stately model of breakthrough, where architectural impact is achieved through monumentality, visual presence, and institutional representation.

In contrast, the Chinese Schools project demonstrates a different mode of breakthrough, primarily expressed through functional and spatial transformation. The project achieves high performance in functional and spatial quality, supported by efficient spatial organization, adaptability, and improved user experience. Its approach is characterized by modularity, repetition, and scalability, enabling rapid implementation across multiple sites. This reflects a programmatic-pragmatic model of breakthrough, where innovation is driven by systemic efficiency and responsiveness to societal needs rather than formal experimentation.

Across all cases, sustainability and technological integration are present but vary in their role and depth. In landmark projects, technology primarily supports structural ambition and formal expression, while in the Chinese Schools project, it contributes to construction efficiency and operational performance. Similarly, contextual interaction manifests differently, ranging from symbolic reinterpretation of identity in landmark projects to functional responsiveness in educational infrastructure.

The variation between the landmark governmental projects and the Chinese Schools project is analytically intentional, enabling the identification of multiple signatures of architectural breakthrough rather than restricting the concept to iconic architecture alone. This comparative approach demonstrates that breakthrough in the Iraqi context can emerge both through formal-symbolic transformation and through functional-systemic innovation.

### Consolidated quantitative evaluation

Applying the scoring framework detailed in the Quantitative scoring framework for case study comparison subsection, which triangulates field observations, document analysis, and interview data, a comparative quantitative assessment of the three case studies was conducted. To ensure a systematic and granular assessment, each case study was evaluated against the specific sub-indicators defined in the analytical framework (Table 1). The detailed scoring for each sub-indicator, derived from the triangulation of observational, documentary, and interview data, is presented in Table 4. Table 5 then synthesizes these results by presenting the arithmetic mean score for each of the four main indicators, providing a consolidated overview of each project’s performance profile. Tables 4 and 5 provide detailed and consolidated representations of the quantitative evaluation, replacing earlier preliminary summaries. The quantitative scores presented in Tables 5 and 6 are derived from the qualitative evaluation of each case study, using the predefined scoring framework described in the methodology. These values are intended to summarize and compare findings rather than to replace qualitative interpretation.

**Table 5:** Detailed Assessment of Case Studies Against Analytical Sub-Indicators.

Main Indicator	Sub-Indicator	General Secretariat Building	Chinese Schools Project	Central Bank of Iraq Building
**1. Design Innovation**	Formal novelty & non-traditional geometry	5	3	5
	Experimental structural systems & techniques	5	2	5
	Rejection/radical reinterpretation of compositional rules	5	3	4
**2. environmental Sustainability & Tech. Integration**	Use of sustainable/energy-producing materials	4	4	5
	Implementation of energy-efficient systems (lighting, HVAC)	4	4	5
	Adoption of innovative construction technologies & smart systems	3	3	5
**3. Interaction with Context & Cultural Identity**	Integration of cultural/heritage symbols with contemporary interpretation	5	3	4
	Use of local or context-sensitive materials and motifs	4	2	4
	Responsiveness to social character and urban morphology	4	4	3
**4. nctional & Spatial Quality**	Spatial efficiency and clarity of user circulation	4	5	4
	Flexibility and adaptability of spaces for varied uses	3	5	3
	Contribution to user comfort, well-being, and quality of life	5	5	4

*Note: Scoring based on the 5-point ordinal scale (1 = Absent, 5 = Exceptional) applied through triangulation of field observation, document analysis, and interview data, as per the rubric in Table 3.

Comparative analysis reveals two distinct models of architectural breakthrough. The first, observed in the General Secretariat and Central Bank projects, represents a symbolic-stately model characterized by formal expression and institutional identity. The second, represented by the Chinese Schools project, reflects a programmatic-pragmatic model, where breakthrough is achieved through functional efficiency and social impact.

**Fig 21:**
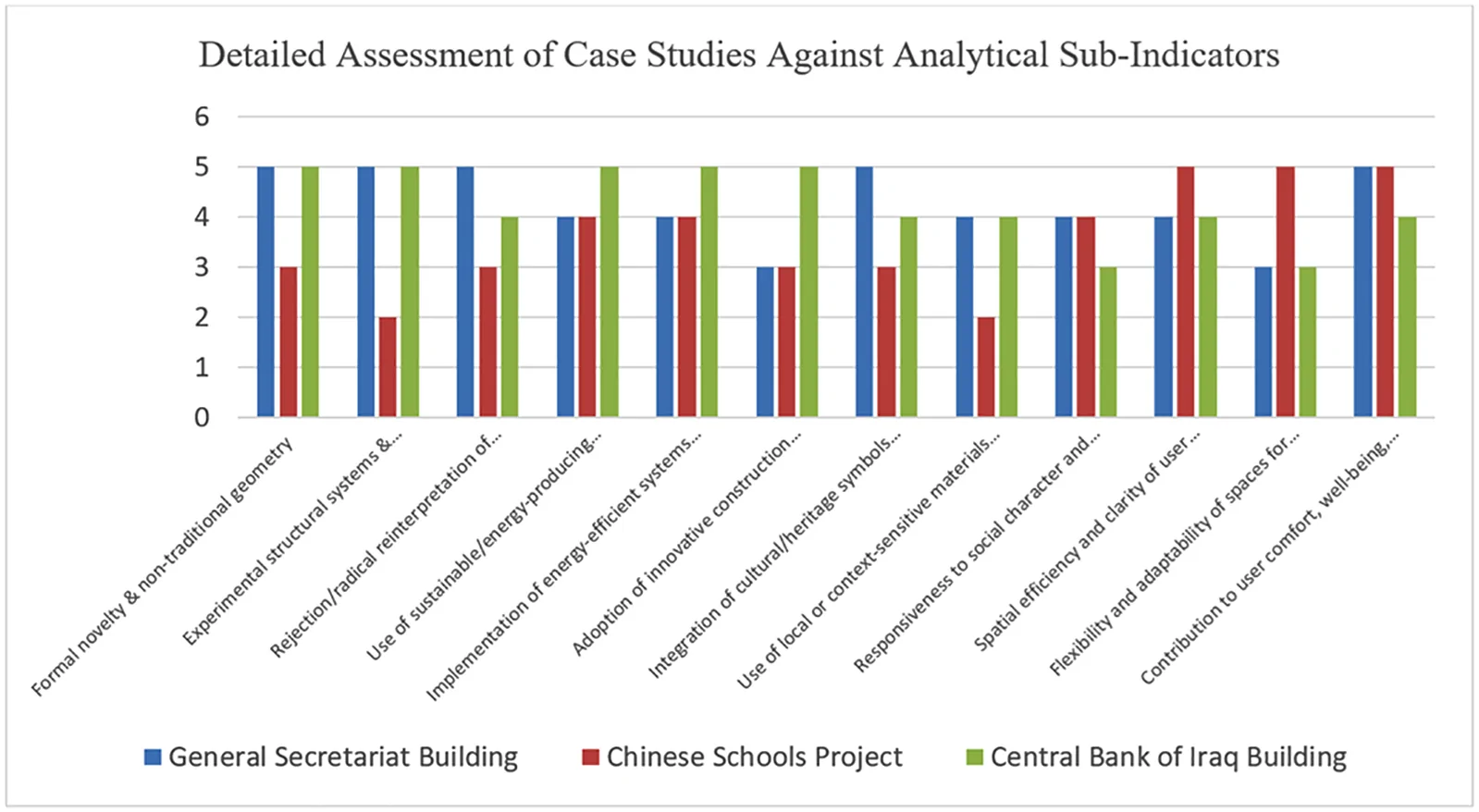
A chart showing illustrating a comparison of the performance of the three case studies across the sub-indices. (create by authors).

To provide a holistic visual comparison of the three case studies’ performance profiles across the four analytical indicators, a radar chart was generated based on the quantitative scores from Table 3. This synthesis moves beyond tabular data to reveal the distinct “breakthrough signatures” of each architectural project, as illustrated in (Fig 21).

### Key qualitative themes from interviews

“Thematic analysis of the interview transcripts yielded several overarching themes that elucidate the drivers, constraints, and perceptions of breakthrough in the Iraqi context. The analysis revealed that institutional frameworks are often perceived as the primary barrier to innovation, while elite sponsorship emerged as a critical enabler for landmark projects. Furthermore, the depth of cultural integration, whether conceptual or ornamental, was identified as a key differentiator in the perceived success of a breakthrough. The most salient themes, their prevalence among participants, and illustrative data excerpts are consolidated in Table 7 below for a comparative overview.”

**Table 7:** Summary of Key Themes from Thematic Analysis of Interviews.

Theme	Prevalence	Representative Quote (Participant)	Brief Interpretation
**Institutional Barriers as Primary Constraint**	9 out of 10 interviewees	*“The biggest designer in Iraq is often the rulebook, not the architect. Innovation must pass through layers of committees afraid of deviation.”* (P7, Academic)	Highlights a perceived systemic obstacle to breakthrough.
**Elite/State Sponsorship as Key Enabler**	Highlighted for landmark projects	*“A project like the Secretariat, with its daring form, would not happen without direct, high-level political backing. It was a top-down vision.”* (P3, Architect)	Identifies a critical driver for achieving formal/aesthetic breakthrough.
**Heritage as Conceptual vs. Ornamental**	Noted in successful identity integration	*“The breakthrough in the Secretariat is that the ancient seal is not applied as a decoration; it is architecture. The form is generated from it.”* (P10, Academic)	Distinguishes between deep conceptual engagement and superficial application of cultural motifs.
**Sustainability as Symbolic Leadership**	7 out of 10 interviewees	*“The Central Bank aiming for BREEAM is more than engineering. It’s a message: ‘We are back, and we are modern.’ It sets a new benchmark.”* (P8, Urban Planner)	Positions sustainability as a tool for institutional and national image-making.
**Breakthrough in User Experience**	Emphasized for the Schools project	*“The real breakthrough of the new schools is in the child’s daily experience—the light, the color, the connection to courtyards. It changes the feeling of learning.”* (P5, Academic)	Shifts the focus of breakthrough from object to experience, from architect to user.

## Discussion

### Synthesizing breakthrough signatures and their implications

The integrated analysis of the three case studies—through descriptive examination, quantitative scoring, and qualitative themes—reveals that architectural breakthrough in contemporary Iraq is not a monolithic phenomenon. Instead, it manifests through distinct “breakthrough signatures,” each shaped by a specific confluence of project typology, intent, and contextual drivers. This synthesis moves beyond cataloguing innovations to propose a contingent understanding of how transformative change is achieved in a complex, post-conflict environment.

### Deconstructing the breakthrough signatures

1 **The Symbolic-Stately Signature (General Secretariat & Central Bank):**

This signature is defined by high-stakes, institutionally backed projects aiming for national and international recognition. Breakthrough here is driven by:

- Elite/State Sponsorship: A top-down mandate for iconic expression, which overcomes typical bureaucratic inertia (Theme: *Elite Sponsorship as Key Enabler*).
- Formal & Technological Ambition: Prioritizing non-traditional geometry and advanced environmental systems as statements of modernity and progress (Indicator: *Design Innovation & Sustainability* scored highly).
- Heritage as Conceptual Generator: The most successful aspect, where cultural identity is not appliqued but becomes the DNA of the form, as in the Secretariat’s cuneiform-inspired massing (Theme: *Heritage as Conceptual vs. Ornamental*).
- Practical Implication: Major architectural landmarks in Iraq may emerge, where political will is a prerequisite for formal and technological experimentation.

2 **The Programmatic-Pragmatic Signature (Chinese Schools):**

This signature emerges from large-scale infrastructural programs focused on societal needs. Its breakthrough is subtler but profound:

- *Breakthrough in User Experience*: Transformation occurs at the human scale, through daylight, color, and spatial organization that redefine the everyday experience of learning (Theme: *Breakthrough in User Experience*).
- Systemic Functional Innovation: The breakthrough lies in the systematic overhaul of a standard building type, prioritizing operational efficiency, psychological well-being, and community space over iconic form (Indicator: *Functional & Spatial Quality* scored highest).
- Contextual Adaptability over Symbolic Dialogue: While modern, the designs show less deep engagement with specific Iraqi heritage, opting for a generic, “internationally modern” aesthetic that ensures replicability (Indicator: *Interaction with Context & Identity* scored lower).
- Practical Implication: Widespread qualitative improvement may be achieved through evidence-based, user-centric principles.

### Cross-cutting insights for the Iraqi context

The study underscores two pivotal, cross-cutting factors that enable or constrain breakthrough across both signatures:

- The Central Constraint: Institutional Inertia. The most frequently cited barrier was a regulatory and approvals culture averse to risk, effectively making “the rulebook the biggest designer” (Theme: *Institutional Barriers*). This highlights a critical tension between aspirations for innovation and the realities of governance.
- The Foundational Criterion: Authentic Identity Integration. Projects that achieved higher critical acclaim and perceptual breakthrough were those where cultural elements were generative (source of form) rather than decorative. This distinguishes a deep architectural dialogue from superficial styling.

### Toward a strategic framework for practitioners and policymakers

For architects, planners, and stakeholders aiming to foster architectural breakthrough in Iraq or similar contexts, the findings suggest a strategic, contingency-based approach:

1. **Align Project Strategy with Signature:** Recognize which “signature” a project aligns with—Is it a state-sponsored icon or a large-scale social program? - and articulate its value proposition accordingly (symbolic stature vs. functional excellence).
2. **Cultivate Patronage for Ambition:** For transformative iconic projects, securing and educating high-level patrons is as crucial as the design itself, to shepherd innovation through complex approval processes.
3. **Leverage Scale for Systemic Change:** In programmatic projects, direct innovative energy towards rethinking user experience, modularity, and sustainability at the systemic level, where impact is multiplied.
4. **Navigate Constraints with Compelling Narratives:** To overcome institutional barriers, architects must build robust, culturally grounded narratives that justify innovation, framing it not as a deviation but as a responsible engagement with heritage and future needs.

In conclusion, breakthrough in contemporary Iraqi architecture is achievable but follows distinct, context-dependent pathways. Understanding these pathways—the Symbolic-Stately and the Programmatic-Pragmatic—provides a strategic map for translating the aspiration for a renewed architectural identity into tangible, groundbreaking reality.

### Limitations of the study

This study has a number of limitations. This study has certain limitations.

The results of the present study must be interpreted with the following limitations in mind: The first limitation of the qualitative investigation was that it was exploratory using only a small case study sample of ten subjects; this may not reflect the diversity of views. Secondly, the respondents were mainly architects, academics and planners, with only one facility manager representing direct users. For the Chinese Schools project, there were no students or educational users involved in the interviews, which is a limitation in evaluating the user experience from the point of view of the main beneficiaries of the educational environment.

Third, it is not always possible to send interview transcripts back to participants for verification or review (member checking); this may constrain the chance for participants to validate the interpretation of the information. Lastly, the study is not representative or generalizable to all contemporary architectural production in Iraq, since only three selected architectural projects are discussed. The findings should be rather interpreted as analytically transferable insights, which adds to the theory of architectural breakthrough in the architectural and institutional context.

## Conclusions

This study aimed to examine the phenomenon of architectural breakthrough in the post-2003 Iraq, with the following fundamental questions: What is an architectural breakthrough? How is it realized? What are the enabling factors for architectural breakthrough in the post-2003 Iraq? Based on a comparative qualitative analysis of the three well-known works of Baghdad, some preliminary conclusions are drawn that can help the theoretical discussion and application to the Iraqi context.

### Defining and measuring breakthrough: A four-pillar framework

This research proposes the potential of operationalizing a concept of “architectural breakthrough”, which could be achieved by creating a framework for analytical structuring containing 4 measurable indicators: Design Innovation, Environmental Sustainability and Technological Integration, Interaction with Local Context and Cultural Identity, and Functional and Spatial Quality. The assessment of the analysed cases through a framework of evidence-based scoring shows that real breakthroughs could be multidimensional and go beyond formal, technical, cultural, and experiential aspects of the process. However, it is important that this framework is applied in other settings to validate it.

### Manifestations of breakthrough: Two distinct signatures

The three cases analyzed indicate that there is no single template for breakthrough, but that it can occur in two different “signatures” that have distinct project typologies and drivers in the examined context:

- The Symbolic-Stately Signature (as seen in the General Secretariat and Central Bank of Iraq): Formal geometry of aspirations, high technical integration, and thoroughness in the understanding of cultural heritage (cuneiform-inspired massing in the Secretariat). In those cases studied, this signature was linked to patronage from the state or the elite.
- The Programmatic-Pragmatic Signature (as seen in the Chinese Schools Project): Focuses on systemic innovation of user experience, functional efficiency and psychological well-being. The key is its approach to a conventional building type at scale, implying that “radical” formal expression need not be the only way to make a transformational impact, if it is at all.

The observations made are only for the cases studied and other studies are required to confirm that similar signatures are observed in other projects in Iraq.

### Enablers, constraints, and the Iraqi context

Institutional inertia is seen by 9 out of 10 participants as the main obstacle to breakthrough in the context analysed, as revealed in the interviews. In contrast, the presence of ‘elite sponsorship’ was found to be a major facilitator for landmark projects in the case studies. The results also indicate that projects with higher Breakthrough evaluations were those in which the cultural identity was not applied superficially, but was integrated conceptually, which is something that should be studied in other projects.

### Theoretical and practical contributions

This is a theoretical contribution to the discussion on innovation in architecture, as the “breakthrough signatures” model is presented as a preliminary analytical scheme that is in opposition to monolithic architectural theories about innovation. The results indicate that the concept of breakthrough may be more of a strategy, dependent on context, than an ideal, but this does need to be tested in other contexts.

In practice, the research provides an initial set of recommendations and questions for architects and policymakers on how to align project strategy with project signature, and either to promote patronage for symbolic landmarks or to invest in user-driven, systemic innovation for programmatic buildings. The results also reveal the urgent need to overcome the institutional obstacles and create a more favorable environment for architectural experimentation in Iraq.

## Supporting information

S1 File COREQ (COnsolidated criteria for REporting Qualitative research) Checklist. (PDF)
