# Supplementary material for: Breakthrough in architecture—Contemporary Iraqi architecture case study
Source: PLoS One. 2026 Aug 31;21(8):e0355155. doi: 10.1371/journal.pone.0355155 (PMC13529224; doi:10.1371/journal.pone.0355155)
Supplement: Supplementary file 1 [file pone.0355155.s001.pdf]

COREQ (COnsolidated criteria for REporting Qualitative research) Checklist

A checklist of items that should be included in reports of qualitative research. You must report the page number in your manuscript where you consider each of the items listed in this checklist. If you have not included this information, either revise your manuscript accordingly before submitting or note N/A.

Topic	Item No.	Guide Questions/Description	Reported on Page No.
**Domain 1: Research team and reflexivity**			
Personal characteristics			
Interviewer/facilitator	1	Which author/s conducted the interview or focus group?	N/A
Credentials	2	What were the researcher's credentials? E.g. PhD, MD	N/A
Occupation	3	What was their occupation at the time of the study?	25
Gender	4	Was the researcher male or female?	N/A
Experience and training	5	What experience or training did the researcher have?	25
Relationship with participants			
Relationship established	6	Was a relationship established prior to study commencement?	25
Participant knowledge of the interviewer	7	What did the participants know about the researcher? e.g. personal goals, reasons for doing the research	N/A
Interviewer characteristics	8	What characteristics were reported about the inter viewer/facilitator? e.g. Bias, assumptions, reasons and interests in the research topic	25
**Domain 2: Study design**			
Theoretical framework			
Methodological orientation and Theory	9	What methodological orientation was stated to underpin the study? e.g. grounded theory, discourse analysis, ethnography, phenomenology, content analysis	23-24
Participant selection			
Sampling	10	How were participants selected? e.g. purposive, convenience, consecutive, snowball	22
Method of approach	11	How were participants approached? e.g. face-to-face, telephone, mail, email	22
Sample size	12	How many participants were in the study?	21-22
Non-participation	13	How many people refused to participate or dropped out? Reasons?	N/A
Setting			
Setting of data collection	14	Where was the data collected? e.g. home, clinic, workplace	22
Presence of non-participants	15	Was anyone else present besides the participants and researchers?	N/A
Description of sample	16	What are the important characteristics of the sample? e.g. demographic data, date	21-23
Data collection			
Interview guide	17	Were questions, prompts, guides provided by the authors? Was it pilot tested?	21-22
Repeat interviews	18	Were repeat inter views carried out? If yes, how many?	22
Audio/visual recording	19	Did the research use audio or visual recording to collect the data?	22
Field notes	20	Were field notes made during and/or after the inter view or focus group?	21
Duration	21	What was the duration of the inter views or focus group?	22-23
Data saturation	22	Was data saturation discussed?	23
Transcripts returned	23	Were transcripts returned to participants for comment and/or	23

Topic	Item No.	Guide Questions/Description	Reported on Page No.
		correction?	
**Domain 3: analysis and findings**			
Data analysis			
Number of data coders	24	How many data coders coded the data?	25
Description of the coding tree	25	Did authors provide a description of the coding tree?	N/A
Derivation of themes	26	Were themes identified in advance or derived from the data?	24
Software	27	What software, if applicable, was used to manage the data?	23
Participant checking	28	Did participants provide feedback on the findings?	23, 49
Reporting			
Quotations presented	29	Were participant quotations presented to illustrate the themes/findings? Was each quotation identified? e.g. participant number	28-31, 45
Data and findings consistent	30	Was there consistency between the data presented and the findings?	28-45
Clarity of major themes	31	Were major themes clearly presented in the findings?	44-45
Clarity of minor themes	32	Is there a description of diverse cases or discussion of minor themes?	31-44

Developed from: Tong A, Sainsbury P, Craig J. Consolidated criteria for reporting qualitative research (COREQ): a 32-item checklist for interviews and focus groups. *International Journal for Quality in Health Care*. 2007. Volume 19, Number 6: pp. 349 – 357

Once you have completed this checklist, please save a copy and upload it as part of your submission. DO NOT include this checklist as part of the main manuscript document. It must be uploaded as a separate file.
